# Functional characterization of plant specific *Indeterminate Domain* (IDD) transcription factors in tomato (*Solanum lycopersicum* L.)

**DOI:** 10.1038/s41598-024-58903-0

**Published:** 2024-04-05

**Authors:** Sujeevan Rajendran, Yu Mi Kang, In Been Yang, Hye Bhin Eo, Kyung Lyung Baek, Seonghoe Jang, Assaf Eybishitz, Ho Cheol Kim, Byeong Il Je, Soon Ju Park, Chul Min Kim

**Affiliations:** 1 Department of Horticulture Industry, Wonkwang University, Iksan, 54538 Republic of Korea; 2 Department of Horticultural and Life Science, Pusan National University, Milyang, 50463 Korea; 3 World Vegetable Center Korea Office (WKO), Wanju-gun, Jeollabuk-do 55365 Republic of Korea; 4 World Vegetable Center, P.O. Box 42, Tainan, 74199 Shanhua Taiwan; 5 Division of Applied Life Science (BK21 Four), Plant Molecular Biology and Biotechnology Research Center (PMBBRC), Gyeongsang National University, Jinju, Korea

**Keywords:** Tomato, Transcription factor, Indeterminate Domain, Development, Stress response, Plant development, Plant molecular biology, Plant stress responses

## Abstract

Plant-specific transcription factors (TFs) are responsible for regulating the genes involved in the development of plant-specific organs and response systems for adaptation to terrestrial environments. This includes the development of efficient water transport systems, efficient reproductive organs, and the ability to withstand the effects of terrestrial factors, such as UV radiation, temperature fluctuations, and soil-related stress factors, and evolutionary advantages over land predators. In rice and *Arabidopsis*, *INDETERMINATE DOMAIN* (*IDD*) TFs are plant-specific TFs with crucial functions, such as development, reproduction, and stress response. However, in tomatoes, *IDD* TFs remain uncharacterized. Here, we examined the presence, distribution, structure, characteristics, and expression patterns of *SlIDD*s. Database searches, multiple alignments, and motif alignments suggested that 24 TFs were related to *Arabidopsis IDD*s. 18 *IDD*s had two characteristic C2H2 domains and two C2HC domains in their coding regions. Expression analyses suggest that some *IDD*s exhibit multi-stress responsive properties and can respond to specific stress conditions, while others can respond to multiple stress conditions in shoots and roots, either in a tissue-specific or universal manner. Moreover, co-expression database analyses suggested potential interaction partners within *IDD* family and other proteins. This study functionally characterized *SlIDD*s, which can be studied using molecular and bioinformatics methods for crop improvement.

## Introduction

In the past five decades, the global population has increased by four billion and is predicted to increase rapidly from the current eight billion individuals^1^. The reduction of arable land and the water crisis in agriculture will be a great challenge in the future^2^. Climate change projections indicate that intense rains will cause floods and long droughts, reducing cultivation periods in the future^3^. Increase in global population, reduction in arable land, and reduction in cultivation periods will exponentially increase the need for intensive farming methods and new crop varieties. Currently, widespread plant breeding methods are likely to limit yield limitation in the near future. Therefore, plant breeders are obliged to discover new tools and principles to increase crop yield.

Owing to their sessile nature, plants have evolved to withstand and counteract biotic and abiotic stress^4,5^. Stress signals from unfavorable conditions, such as temperature, waterlogging, drought, oxidative stress, proton stress, heavy metals, salinity, light, viruses, bacteria, fungi, and insects are perceived by receptor complexes, and the perceived signals are transduced to TFs to activate stress response genes^6,7^. TFs interact with the *cis*-regulatory elements of a target gene and modulate its expression of their target genes^8^. Changes in *cis*-regulatory elements result in alterations in target gene expression, which can alter cellular activities^9–12^. TFs sequences specifically bind to transcription factor binding motifs (TFBMs) to activate or repress downstream genes with a DNA-binding domain^13,14^. TFs also contain oligomerization, transcription, and nuclear localization domain^15^. Changes in the domain architecture of TFs can be a driving force in plant evolution and changes in the expression can result in morphological variations^16,17^. Plant-specific TFs regulate genes related to the development of plant-specific organs and response systems for adaptation to terrestrial environments^18^. These include the development of efficient water transport systems, efficient reproductive organs, the ability to withstand the effects of terrestrial factors such as UV radiation, temperature fluctuations, soil-related stress factors, and evolutionary advantages over land predators^19–21^. *INDETERMINATE DOMAIN* (*IDD*) TFs are plant-specific TFs with crucial functions in rice and *Arabidopsis*, including development, reproduction and stress response^22–28^.

Among the vast array of TFs, *IDD*, a class of C2H2 zinc-finger TFs, is specific to plants^22,25,29,30^. The N-terminus of the *IDD* contains two C2H2 DNA-binding domains and 2C2HC protein-binding domains. The C-terminus also contains protein interaction domain^24,25,29^. In *Arabidopsis*, 12 of 18 identified *IDD* TFs have been characterized for their roles. *IDD*s in Arabidopsis are involved in various cellular and developmental functions such as seed germination, tissue patterning, responses to external cues, and abiotic stress. Some *IDD*s can produce transcript variants, depending on the conditions (see review)^22^.

Tomatoes (*Solanum lycopersicum*) are one of the most cultivated crops in the fresh and processed market. Owing to its relatively small genome size and chromosomal architecture, the tomato is also an excellent model plant for studying Solanaceae species^31,32^. Tomatoes also bear berry fruits, which can be used as models for studying fruit development and metabolite analysis^33–35^. Studies on the abiotic and biotic responses in tomatoes have been widely conducted. To understand the *IDD* family genes in tomato (*SlIDD*s), this study was conducted to identify and explore the basic information of *SlIDD*s and to understand their expression dynamics under developmental stages and stress conditions in tomato.

## Results

### Identification and phylogenetic analysis of *SlIDD* family genes in tomato

**Figure 1:**
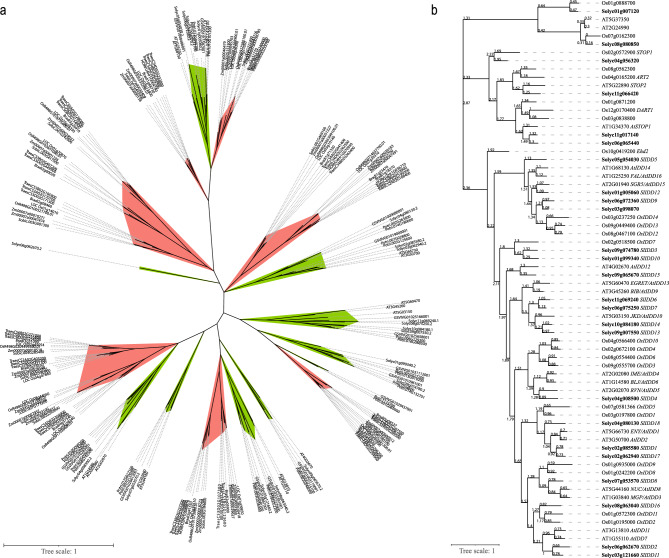
Phylogenetic analyses of *IDD* family genes in major plant species. (**a**) Unrooted phylogenetic tree of *IDD* family genes in ten major plant species. Red branches indicate monocots and green indicate dicots. (**b**) Phylogenetic tree of *IDD* family genes in rice, Arabidopsis, and maize. Tomato sequences are indicated in bold letters [branch values (MYA)].

To identify candidate Sl*IDD* family genes, a BLAST search was conducted using Gramene (https://www.gramene.org) and Plaza (https://bioinformatics.psb.ugent.be/plaza) databases. Overall, 25, 24, and 20 genes were identified by search results in tomato, rice, and *Arabidopsis* respectively. *Arabidopsis* and rice have 16 and 15 *IDD* genes, respectively. The evolutionary relationships among *IDD* family genes were determined using phylogenetic analysis. Phylogenetic analysis suggested that *IDD* genes may have structural differences between monocots and dicots (Fig. 1a). Four subgroups of *IDD-like* genes have been identified in tomato plants. Here, 16 Arabidopsis and 15 rice *IDD* genes were clustered with 19 tomato *IDD*-like genes. Among these clades, rice *Ehd2* showed the lowest homology with other *IDD* genes. 12 genes clustered with the At*STOP1* group and seven genes showed distant homology with *IDD* genes (Fig. 1b).

### Structure and distribution of *SlIDD* genes

**Figure 2:**
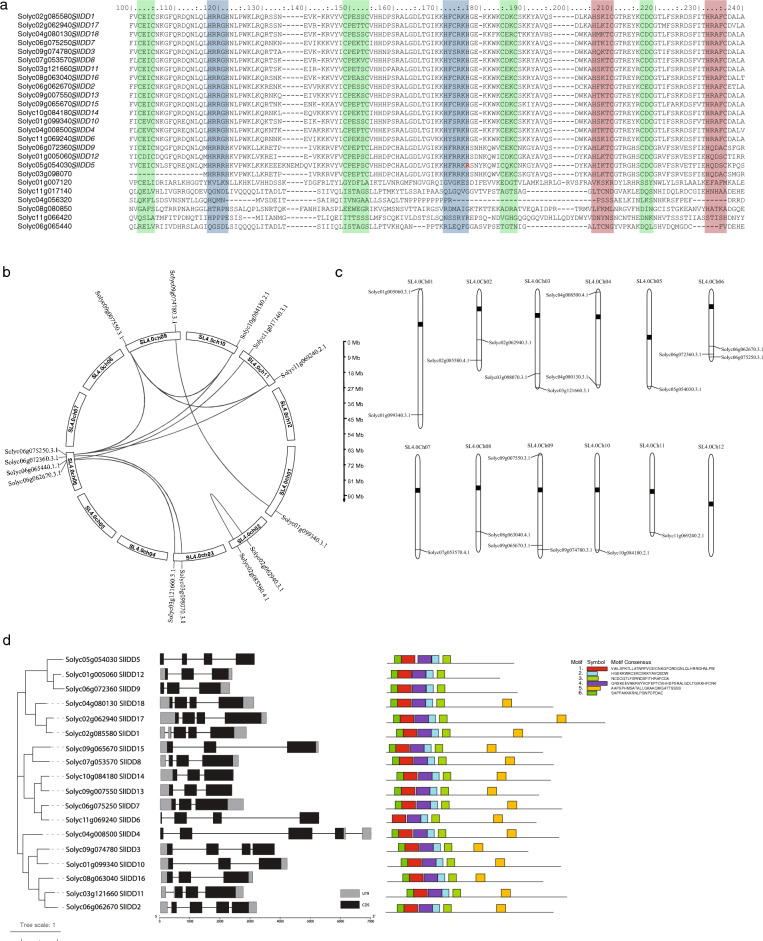
Comparison, confirmation, and distribution of tomato *IDD* sequences. (**a**) Multiple alignment of *IDD*-like genes in tomato. (green; cystine motifs, Blue; histidine motifs, Red; histidine–cystine motifs. Red bold R indicates the arginine in the C2HR motif). (**b**) The synteny analysis of the SlIDD family in tomato. The genes linked by red lines represent homologs. (**c**) Distribution of 18 *IDD* TFs in tomato chromosomes (Black lines indicate synteny). (**d**) Phylogenetic relationship and gene structure of 18 confirmed *IDD* genes (left) and protein motifs in corresponding sequences (right).

Multiple sequence alignments showed conserved C2H2 and C2HC motifs among *SlIDD* genes (Fig. 2a, Fig. S1). However, Solyc03g098070 does not possess the first C2H2 motif. Solyc05g054030 possesses a C2HR motif in the second zinc finger domain with a less reactive arginine stead of Histidine^36^ (Fig. 2a). seven ortholog groups were found within tomato IDD-like genes. Block 6 and block 7 contained four and three orthologs respectively (Fig. 2b and Table S1). Among them Solyc01g005060, Solyc04g080130, Solyc04g008500, Solyc05g054030, Solyc07g053570, Solyc08g063040 and Solyc09g065670 did not show orthologs. Synteny between Arabidopsis, rice and tomato revealed that the rice *IDD* family showed the least synteny when compared with Arabidopsis and tomato (Fig. S2). Sequences with two complete C2H2 complete C2HC domains were considered as true *IDD* TFs. After confirming the number of *IDD* genes in tomatoes, the distribution of *IDD* genes were determined (Fig. 2c, Table S2). Twelve *IDD*s showed synteny, indicating duplication events in all chromosomes. Dispersed duplications were accounted for the majority (80%) of *IDD* like genes and other genes were duplicated by segmental duplication events (Table S3). Solyc03g098070, which lacks the first C2H2 motif, exhibited synteny with Solyc06g072360. 18 confirmed *IDD* genes were distributed among 11 chromosomes, excluding chromosome 12. Motif analysis revealed structural variations among *IDD*-like genes. Among the 25 sequences, 18 *IDD* TFs had four prominent motifs corresponding to two C2H2 and two C2HC domains in the C-terminus. Other *IDD*-like genes lacked one or more zinc-finger domains (Fig. 2d and Fig. S3). Ka/Ks analysis revealed that all *IDD* genes evolved under high selection pressure (Table S4). In addition to the primary isoforms of *IDD* TFs, our analyses revealed that multiple *IDD*s have splice variants. *IDD1*, *IDD2*, *IDD12* and *IDD13* showed two isoforms. Surprisingly, *IDD4* and *IDD11* had three and five isoforms, respectively, indicating complex post-transcriptional regulatory mechanisms present in *IDD* TFs (Table S5).

### 3D structure of *SlIDD* TFs

**Figure 3:**
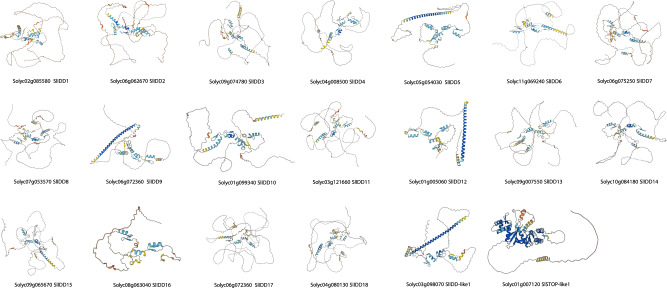
3D models of tomato *IDD* TFs predicted by AlphaFold2.0. Light blue chains show zinc finger domains. 2D images were taken for visibility of zinc finger domains (see Table S6 for AlphaFold2.0 accession numbers to access 3D models).

Following the identification of *SlIDD* TFs, 3D structures were predicted using AlphaFold2.0 (https://alphafold.ebi.ac.uk) to verify the structural similarity of the confirmed TFs using BLAST with UniProt (https://www.uniprot.org) accession numbers (Table S6). The 3D structures showed prominent zinc finger domains in the C terminus regions of the primary isoforms. However, Solyc03g098070 had only three zinc finger domains, which confirmed the results from motif analysis and multiple alignments, and Solyc08g063040 showed an incomplete 4th C2HC domain, even though the alignments and motifs were intact (Fig. 3).

### Cis-regulatory element analysis of *SlIDD* promoters

**Figure 4:**
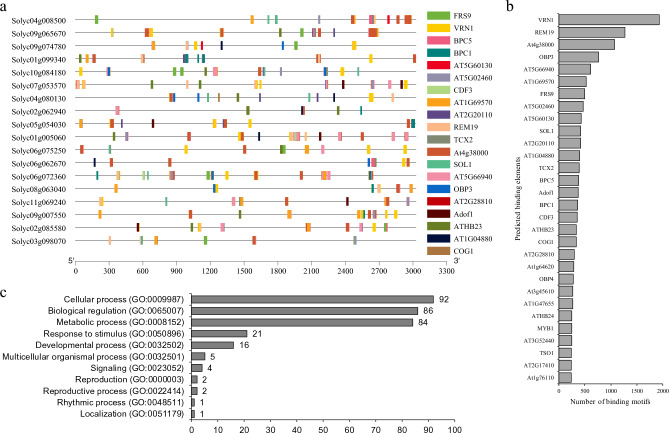
*Cis*-regulatory element analysis of *SlIDD* family genes. (**a**) Promoter binding sites of tomato *IDD* TFs. (**b**) Enriched promoter binding elements in tomato *IDD* TFs (50% of enriched elements). (**c**) GO term analysis for enriched promoter elements.

The promoter sequences of 18 *SlIDD* genes (3000 bp) were scanned for *cis*-regulatory elements using Arabidopsis DAP motifs with a cut-off p-value of 1 × 10^−4^. A total of 518 binding elements were present in all 18 promoter sequences, and *VRN1*, *REM19,* and *DOF4.7* binding elements were relatively more enriched (14.65%) than other promoters (Fig. 4a, b and Table S7). Most of the enriched elements showed functions related to environmental signal response and development (Fig. 4c).

### Interaction networks of *SlIDDs*

**Figure 5:**
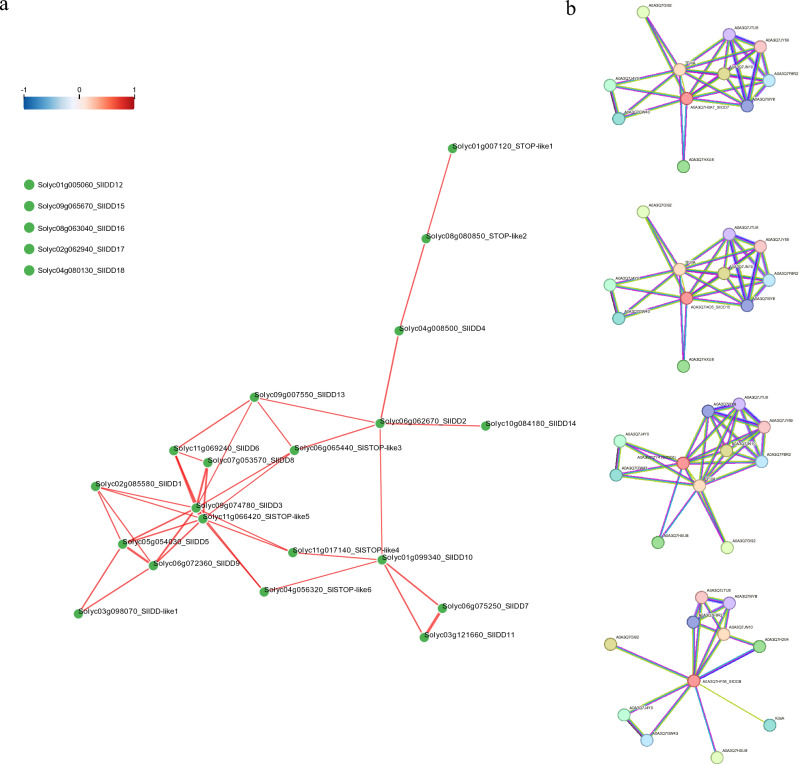
Coexpression networks for *SlIDD*s. (**a**) Coexpression network between *SlIDD*s, *SlIDD-like1,* and *STOP1-like TF*s. (**b**) Interaction network of *SlIDD6*, *SlIDD7*, *SlIDD15,* and *SlIDD8* with other proteins.

Coexpression network analysis revealed complex interactions between *SlIDD*s and *STOP-like* TFs. Unlike tissue expression patterns, co-expression networks suggested possible differences in temporal expression patterns and provided clues to gene regulation networks in different tissues (Fig. 5). *SlIDD4* showed close association with *SlIDD2* similar to the tissue-specific expressions. However, *SlIDD12* did not interact with other *SlIDD*s, including *SlIDD15*, *SlIDD16*, *SlIDD17,* and *SlIDD18*. *SlIDD3* showed multiple interactions with the other *SlIDDs*. *SlIDD10* interacted with *SlIDD2*, *SlIDD7,* and *SlIDD11*; however, the tissue-specific expression patterns were distant from those of *SlIDD11*. *SlIDD13* and *SlIDD14* interacted with *SlIDD2* but showed similar expression patterns in tissues. Compared to other interactions, there are less data on *SlIDDs*, therefore, databases have shown that *SlIDDs* are co-expressed with *TFIIIA* and *SlkdsA*.

### Expression of IDD TFs under abiotic stresses

*Cis*-regulatory analysis suggests that the binding elements in the promoters are highly responsive to environmental signals. Moreover, *SlIDD1*, *SlIDD8*, *SlIDD9,* and *SlIDD16* show increased expression under various abiotic stress conditions^37,38^. To confirm whether other *SlIDD*s were also responsive to abiotic stress, 3-week-old plants grown under greenhouse nursery conditions were subjected to salt, pH, and flood stress, which represent the basic stress conditions that can occur under greenhouse conditions (see Materials and Methods). Expression analysis was conducted to determine the expression level of each *SlIDD* TFs.

### Expression of *SlIDD* TFs under salt stress

Salt stress can affect plants by restricting water and nutrient uptake, resulting in reduced root biomass and reduced productivity^39^. Nutrient imbalance owing to NaCl-induced conductivity stress reduces fruit quality under greenhouse conditions^40^. To determine the expression patterns of *SlIDD* TFs, 3-week-old tomato seedlings were treated with 200 mM NaCl and sampled at 2- and 24 h intervals.

**Figure 6:**
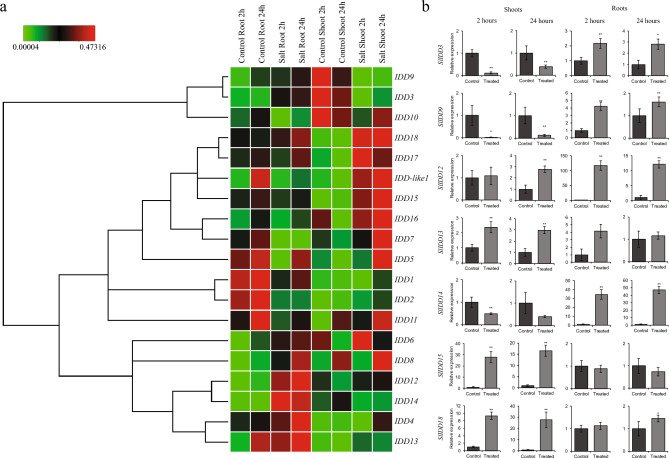
Expression patterns of screened zinc finger TFs under salt stress. (**a**) Clustergram for *IDD* expression levels under NaCl induced salt stress in 3-week-old tomato seedlings (Scales represented as relative values). (**b**) Expression levels of high responsive *SlIDD*s under salt stress. (**P < 0.01; *P < 0.05).

Expression analysis revealed that the levels of multiple *SlIDD*s were upregulated in the roots under salt stress conditions (Fig. 6a). *SlIDD12* and *SlIDD14* showed over 100- and 30-fold increases in expression, respectively, whereas *SlIDD3*, *SlIDD4,* and *SlIDD9* showed only significant increases in expression. Other *SlIDD*s such as *SlIDD1* and *SlIDD2* showed significantly reduced expression. *SlIDD4* and *SlIDD13* were upregulated after 24 h of treatment, whereas *SlIDD6* showed increased expression only after 2 h. However, in the shoots, *SlIDD12* and *SlIDD14* did not show a significant increase in expression, whereas *SlIDD15* and *SlSlIDD18* showed a dramatic increase in expression. Significant increases in expression were observed in *SlIDD13* and *SlIDD17* in 2 h. *SlIDD1*, *SlIDD7*, *SlIDD12*, *SlIDD16*, *SlIDD2*, *SlIDD4,* and *SlIDD-like1* showed significantly higher levels in 24 h. *SlIDD3* and *SlIDD9* showed a significant reduction in expression under salt stress conditions (Fig. 6b and Table S8).

### Expression of *SlIDD* TFs under pH stress

Low pH is an occasional problem in greenhouse vegetable production, as it can affect the quality and quantity of produce by affecting soluble ions in the media^41,42^. *SlSTOP1* is an essential TF that is closely related to *SlIDD* TFs and crucial for proton stress tolerance^43,44^. To examine the expression of *SlIDD* TFs, plants were subjected to low pH conditions (pH = 4.2) in 3-week-old plants.

**Figure 7:**
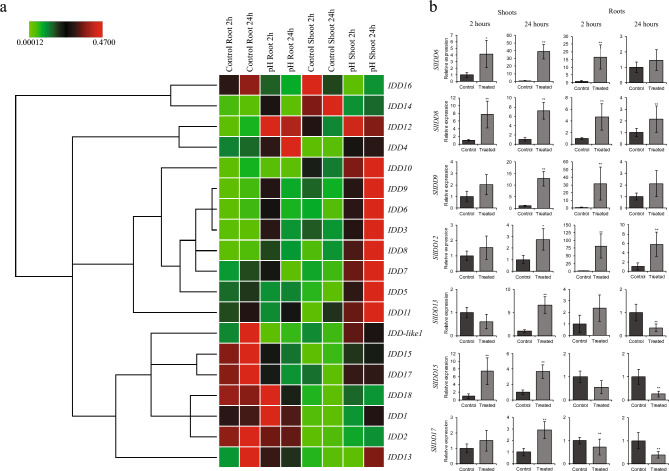
Expression patterns of screened zinc finger TFs under proton Stress. (**a**) Clustergram for *IDD* expression levels under pH stress in 3-week-old tomato seedlings (Scales represented as relative values). (**b**) Expression levels of high responsive *SlIDD*s under pH stress. (**P < 0.01; *P < 0.05).

The expression of *SlIDD12* was up-regulated in the roots by over 50-fold. *Sl*IDD8 also showed a significant increase in the roots (Fig. 7). In contrast, *SlIDDlike-1*, *SlIDD13*, *SlIDD15*, *SlIDD16,* and *SlIDD17* showed significant reductions in expression levels in the roots. However, in the shoots, *SlIDD6* showed a 40-fold increase in expression after 24 h. Notably, *SlIDD15* and *SlIDD17* showed significant increases in the shoots, but not in the roots. *SlIDD8* expression was significantly higher in both tissues at both time points (Fig. 7b and Table S9).

### Expression of *SlIDD* TFs under flooding stress

Flooding stress is a major problem for field-cultivated tomatoes because of the intensive rainfall patterns that induce climate change^45^. Waterlogging reduces oxygen availability to the submerged plant parts, which subsequently leads to cell death and, eventually severe yield losses^46,47^. Because *IDD* TFs are plant-specific, they can potentially respond to flood stress. To determine the response of *IDD* TFs to flood stress, 3-week-old tomato seedlings were submerged in water up to the crown, and RNA was extracted from the roots and shoots at 2 h and 24 h intervals.

**Figure 8:**
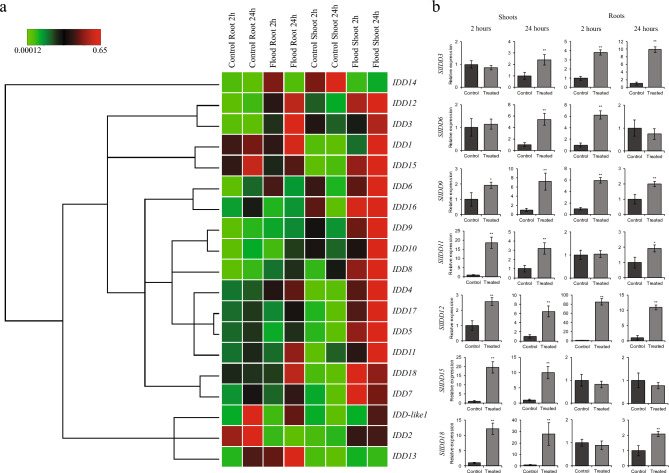
Expression patterns of screened zinc finger TFs under flood stress. (**a**) Clustergram for *IDD* expression levels under flood stress in 3-week-old tomato seedlings (Scales represented as relative values). (**b**) Expression levels of high responsive *SlIDD*s under flood stress. (**P < 0.01; *P < 0.05).

Unlike salt and pH stress, less severe reaction of *SlIDD* TFs were observed in the roots (Fig. 8a and Table S9). Among strongly responded seven genes to flood, *SlIDD12* showed an 80-fold increase in expression. Moreover, *SlIDD3*, *SlIDD6*, *SlIDD9* and *SlIDD18* showed significantly increased expression (Fig. 8b). In contrast, *SlIDD2* was downregulated in roots and upregulated in shoots at 24 h time points. In the shoots, all genes except *SlIDD7*, *SlIDD13,* and *SlIDD14* showed increased expression levels. In particular, *SlIDD18* showed more than tenfold and 28-fold increase at the 2 h and 24 h intervals, respectively. *SlIDD11* and *SlIDD15* also exhibited dramatic increases in the shoots under flood stress (Table S10).

## Discussion

### Functional analysis of SlIDDs

The present study systematically analyzed *IDD* TFs belonging to tomatoes, as *IDD*s in *Arabidopsis*, rice, and maize have already been examined for their existence and properties^22–25,27,48–51^. Currently, there are 16, 15, and 22 *IDD* TFs identified in *Arabidopsis*, rice, and maize, respectively. Consistent with our results for 18 *IDD*s in tomatoes, the *IDD* family genes might have played crucial roles in a species-specific manner. *IDD* TFs are also plant-specific and can participate in multiple plant-specific functions such as vascular development, photosynthesis, light signaling, flowering etc^52^. Moreover, plant-specific TFs are also involved in shaping the phenotypic and physiological factors of plants for the adaptation of plants to land-based environments, where the plants need to withstand biotic and abiotic stress conditions^53^. Functional characterization can shed light on the *IDD* TFs role in plants. Moreover, some plant-specific TFs show differences in the number of monocot and dicot species^54,55^. Phylogenetic analysis of IDD TFs from the model plant *Arabidopsis* and major model crops such as tomato and rice suggested that IDD transcription factors other than higher conservation of their functional motifs in monocots and dicots and structural elements are potentially specialized within each of these two lineages.

Phylogenetic analysis revealed closely related *IDD* groups in rice, tomato, and *Arabidopsis*. *SlIDD1* showed close relationships with *AtIDD1*, *AtIDD2*, *OsIDD1*, *SlIDD17,* and *SlIDD18* (Fig. 1b). *AtIDD1* is involved in gibberellin signaling by forming activator and repressor complexes upstream of gibberellin biosynthesis genes^56^. Notably, *AtIDD1* is a direct target of *PHYTOCHROME INTERACTING FACTOR 3-LIKE5* (*PIL5*), which inhibits seed germination in dark conditions by regulating abscisic acid (ABA) and Gibberellic acid (GA)^57^. *AtIDD2* (*GAF1*) also shows light-responsive properties where *AtIDD2* acts as a transcriptional activator and repressor of *GA20OX* under different light conditions and regulates flowering and plant size^56^. *OsIDD1* along with *OsIDD6* potentially have redundant functions in floral transition^58^. *OsIDD1* also regulates the expression of JA-related genes related to herbivore resistance^59^. *SlIDD1* expression was significantly down-regulated in salt-stressed roots and increased in salt-stressed shoots after 24 h. Under acidic conditions, *SlIDD1* expression was reduced within a short time and recovered after 24 h. Under flooding conditions, the shoots showed significantly higher expression levels, suggesting a pivotal role in the transition under stress conditions (Figs. 6, 7, 8). *SlIDD17* is significantly responsive to salt and acidic stress. Previous studies have shown increased expression of *SlIDD17* under heat stress and during fruit development^60,61^.

*SlIDD2* is closely related to *AtIDD7*, *AtIDD11*, *OsIDD2*, *OsIDD11*, *SlIDD10* and *SlIDD11* (Fig. 1b). *AtIDD7* shows higher activity during phosphorus starvation and flowering^62,63^. In rice, *OsIDD2* negatively regulates the transcription of genes involved in lignin biosynthesis^64^. *OsIDD11* hypothesized to have drought tolerance via stomatal control^65^. In the current study, under stress conditions, *SlIDD2* transcripts were significantly downregulated in the roots and increased in the leaves. *SlIDD2* is down-regulated in the base margin tissue of *tf-2* leaf patterning-deficient mutants^66^. When treated with auxin and ethylene, *SlIDD2* showed increased and decreased activity in fruits, respectively, and reduced expression in *LATERAL ORGAN BOUNDARIES(LOB)* TF and *SlLOB1* RNAi lines, with reduced softening and increased shelf life^67,68^. In Arabidopsis, *AtIDD7* shows differential expression under phosphorus starvation^69^, early flower development^63^, and low temperature^70^. However, the precise function of *AtIDD7* is currently unknown^22^. *AtIDD11* shares structural homology with and is potentially involved in leaf patterning^22,71^. *SlIDD10* showed higher expression levels in maturing fruits and the root exodermis in previous studies^60,61,72^. Interestingly, *SlIDD11* showed a sudden dramatic increase in expression in shoots under all stress conditions, suggesting a role similar to *OsIDD11*. GWAS studies suggest that *SlIDD11* is associated with isothermality and shows allele specificity in exotic land races^73,74^. Notably, *SlIDD11* produced five isoforms that may be expressed under specific stress conditions to respond specifically (Table S5).

*SlIDD3* grouped along with *AtIDD12* and *SlIDD15*. *SlIDD3* showed higher expression patterns in the exodermis and the cortex^72^. *SlIDD15* shows a gradual reduction from young to mature fruits^60^. Under stress, *SlIDD15* showed higher levels of expression in roots, especially under salt stress. *SlIDD3* also showed varying expression patterns in the roots and shoots under stress. *AtIDD12*s function is currently unknown, but it shows higher activity in seeds^75^.

*SlIDD4*, *AtIDD5,* and *AtIDD6* grouped in phylogenetic analysis (Fig. 1b). The Arabidopsis homolog *AtIDD5*/*RAVEN* interacts with *DELLA*^28^ and promotes anisotropic growth by positively regulating *STARCH SYNTHASE 4* (*SS4*)^50^ possibly regulating root tissue patterning through asymmetric cell division^76^. *AtIDD6* is also involved in the tissue patterning of roots during development^77^. However, in tomatoes, stress treatments showed a significant response to salt and acid stress in roots and to flood stress in shoots, suggesting a role in root patterning under both developmental stages and stress tolerance in both roots and shoots (Figs. 6, 7, 8). Notably, *SlIDD4* produces four isoforms (Table S5).

Even though *SlIDD5* contains a C2HR motif instead of a C2H2 motif, evidence suggests that the replacement of Histidine by Arginine might not have any major effect on transcriptional activity (Fig. 2). However, C2HR motifs have been shown to interact with other proteins^36^. *SlIDD5*/*OBV* was highly expressed in the leaves and vegetative phases of the meristems. Heterobaric leaves contains Bundle sheath extensions in the leaves which provide mechanical strength. *SlIDD5* mutants failed to produce bundle sheath extension cells (homobaric leaves)^78^. Increased chlorophyll content has been observed in *obv* mutants, such as M82 and CRISPR/Cas9 mutants of Micro-Tom, where the absence of BSE allowed chloroplast development in leaf veins and reduced water conductivity^79,80^. Moreover, *OBV* also regulates the leaf insertion angle, leaf margin serration, and fruit shape, and has been shown to work together with auxin signaling^80^. *SlIDD5* binds to the promoter *FUL2* which then regulates fruit shape^81^. Arabidopsis *AtIDD14*, *AtIDD15,* and *AtIDD16* show structural homology with *SlIDD5* and have similar functions in leaf shape, flower development, plant architecture, and gravitrophic responses by regulating auxin biosynthesis and transport factors^23^. Surprisingly, *OsIDD14*/*LPA1* also shows similar functions in plant architecture and has been extensively studied. *LPA1* determines rice tiller angle and shoot gravitropism by affecting the sedimentation rate of amyloplasts and binds to the promoter region of *PIN1*^82,83^. *LPA1* also exhibits water conservation properties by reducing the rate of transpiration from rice leaves^84^. However, data for *OsIDD12* and *OsIDD13* were unavailable.

*AtIDD9*, *AtIDD10*, *AtIDD13*, *SlIDD6*, *SlIDD7*, *SlIDD13,* and *SlIDD14* grouped together in the phylogenetic analysis (Fig. 1b). Reduced pH also caused a higher accumulation of *SlIDD6* in shoots (Fig. 6) and showed heat-induced expression in tomato leaves^85^. *SlIDD7* expression patterns were similar to those of *SlIDD10.* Under Salt stress conditions, roots showed reduced expression and shoots showed increased expression. *SlIDD7* expression increases in leaves and stems under heat stress^61^ and is negatively correlated with *CYC-B* in developing fruits, indicating its possible role in the regulation of lycopene accumulation in developing fruits^60^. Both *SlIDD13* and *SlIDD14* regulate stem thickness and leaf shape, and mutants are tolerant to necrotrophic infection^51^. Under salt stress, the roots showed higher levels of *IDD14* transcripts. *IDD13* also showed a significant increase in the shoots of the salt-stressed tomato seedlings (Fig. 8).

*SlIDD8* showed homology with *OsIDD8*, *AtIDD3,* and *AtIDD8* which showed increased expression under salt and heat stress (Fig. 1b)^86^. *AtIDD3* and *AtIDD8* are involved in root development. Moreover, *AtIDD8* regulates floral transition and sugar metabolism^22^.

*SlIDD9* is highly expressed in roots and developing fruits, and shows increased expression under abiotic stress conditions^87^. Arabidopsis *AtSTOP1* shows close homology with *SlIDD9* and is involved in proton toxicity and aluminum tolerance by activating the malate transporter *AtALMT1*^88,89^. *AtSTOP1* also modulates the response to drought and salt levels by regulating root growth and guard cell movement^90^. *SlIDD-like1* showed close homology with *OsLPA1*. Even though *SlIDD-like1* does not contain the first C2H2 domain, it shares close homology with *SlIDD12* and *SlIDD5*. *SlIDD-like1*/*Se3.1* controls stigma extortion or insertion with *Style3.1* which determines the rate of self-pollination^91,92^. Under stressful conditions, *SlIDD-like1* showed less severe changes in expression.

*SlIDD16*/*SlZF-31* mutants showed reduced salt and drought tolerance^38^. *SlIDD16* showed reduced expression in *RIN* mutants, suggesting its potential role in fruit ripening^93,94^. The rice orthologs *SlIDD16, OsIDD11* are involved in drought tolerance by regulating stomatal movement and starch composition in rice^65,95^. *OsIDD2* regulates the secondary cell wall (SCW) formation by directly binding to SCW biosynthesis genes^48^. *OsIDD2* is responsible for plant height, leaf strength, and resistance to fungal infection^64,96^.

In the interaction analysis, more clues and the possible applicability of *SlIDD*s were revealed. *SlIDD6* and *SlIDD7 SlIDD15* showed co-expressed with TFIIIA during viral infection. *Arabidopsis thaliana* experiments have hypothesized that TFIIIA acts as a bridge between the viroid template and DNA polymerase II during viroid-derived RNA replication^97,98^. *SlIDD8* closely interacts with *SlkdsA*, a Kdo-8-P synthase associated with cell division^99^.

Stress experiments revealed that *SlIDD10, SlIDD5, SlIDD7*, *SlIDD13,* and *SlIDD16* showed less dramatic changes in expression, suggesting that these TFs are highly involved in development^51,61,91^. However, *SlIDD16* mutants are tolerant to salt and drought stress, suggesting *SlIDD16* is stress specific^38^. In contrast, *SlIDD3, SlIDD8, SlIDD9,* and *SlIDD12* showed multi-stress responses, suggesting that these TFs should be further studied for their effects on tomato survival and productivity.

### Future perspectives for IDDs for breeding climate-resilient and high-producing crops

The evolution of plants from aquatic to terrestrial habitats is noteworthy. Unlike in aquatic environments, land plants have to increase their survivability by specializing in organs to compartmentalize functions, such as developing effective root systems and vascular systems for water transport, increasing photosynthetic ability and survival adaptations, such as distinguishing beneficial organisms from pathogens and predators, and adapting to dry terrain^100^. Plant-specific transcription factors drive adaptation through genome and gene duplication events and specialize in downstream fuctions^19,21,101,102^.

Climate change threatens crop productivity due to changes in agro-climatic conditions, and current breeding programs are exploring possibilities to develop climate-resilient cultivars for better productivity^103–106^. A better alternative for escaping climate catastrophes without breeding for climate resilience is to cultivate crops in protected environments, such as greenhouses. However, due to the fact that the cultivated crops are highly adapted to pre-climate change era, breeding programs should focus on the evolution of terrestrial plants to identify evolutionarily significant candidate genes for plant breeding.

Evolutionary analyses of *SlIDD* TFs indicated that these genes were selected under high selection pressure and all genes were crucial for survival. In particular, *IDD*s are plant-specific and are involved in functions such as herbivore resistance and starvation responses from germination to fruit ripening. Our current data and those of previous studies show that these *SlIDD*s are potential candidates for improving the productivity of protected house cultivation and land cultivation^60,61,88,89,107^. In the case of land cultivation, *IDD*s respond to abiotic stresses, such as drought, salt, flooding, pH changes, and starvation, along with the development of roots. Other *IDD*s, such as *SlIDD2* showed leaf-patterning roles, and *SlIDD5* showed chlorophyll content, which can be used to increase photosynthetic capacity and productivity. Under changing climates, indoor farming can reduce exposure to harsh climates, which can reduce the energy spent on defense mechanisms^108,109^. However, it is possible to reduce the stress response to eliminate pests and stress in well-protected houses, which renders the stress response elements in plants insignificant^110–112^. Stress response-related genes can be down-regulated to force plants to focus on productivity by diverting energy allocations^112,113^. Finally, the marketability of produce is a crucial factor in increasing the net returns from tomato cultivation^114–116^. *SlIDD*s such as *SlIDD5* and *SlIDD16* showed functions related to fruit shape and ripening, which can be further studied to improve fruit shape and shelf life, and increase market value and post-harvest quality. Plants produce isoforms to diversify their roles by alternative splicing (AS), from a single coding region to multiple protein derivatives for specialized roles^117^. This mechanism allows the plants to eliminate the necessity to harbor additional genetic information in the genome and increase the transcriptome plasticity and proteome complexity^118^. With this mechanism, plants are able to respond against a large array of environmental stresses and cellular damages^119–123^. The isoforms of *SlIDD*s can be further dissected based on their specific roles in growth and development, where a single *SlIDD* responded to various stress conditions in our study (Figs. 6, 7, 8). Studying the role of isoforms can provide insights into the isolation of stress responses and developmental elements from a single TF.

## Conclusions

Amid climate change manifesting in real time, food security must be ensured in every corner of the world. Agro climatic factors may also change with the increase in average global temperature and humans may have to modify crops to ensure cultivation in limited resources and possibly indoors under artificial conditions^124,125^. The current analysis identified 18 *IDD*s (*SlIDDs*) in tomatoes. Functionally, only a few *SlIDD* have been characterized based on molecular evidence. Current study revealed the multi-role potentials of the the *SlIDD* TFs in tomato growth, development and plasticity. Notably, *SlIDD1*, *SlIDD3*, *SlIDD4*, *SlIDD6* to *9*, *SlIDD11*, *SlIDD16* and *SlIDD17* showed potential roles in abiotic stress responses where *SlIDD*s 4 and 11 showed three and five isoforms respectively. Which indicates the functionally diverse role of these TFs. Moreover, previous studies showed *SlIDD13* and *SlIDD14* are involved in abiotic stress response^50,97^. On the other hand, *SlIDD*s *2* to *5*, *SlIDD10*, *SlIDD15*, *SlIDD17* showed potential roles in growth, organ pattering^51,61,91^. These results indicate that the *SlIDD*s are capable of regulating overall plant development, plasticity and physiology in a well-coordinated manner. Based on the results presented here, the functions of *SlIDDs* may be applied well beyond stress tolerance, productivity, and quality of tomato production, where some mutants of *SlIDD*s show crucial agroeconomic traits that can aid in breeding climate-resilient, high-producing tomato cultivars with the aid of the tomato PAN genome. Based on current expression patterns and ortholog functions, embryo lethality is possible. However, other techniques, such as promoter engineering^8,11,12^ or RNAi^126–129^ can be employed to study the molecular functions of *SlIDD*s. Natural disasters and temperature fluctuations have increasingly challenged the future of agriculture. TFs that play a major role in land adaptation can be repurposed to adapt to the current climate crisis, and *SlIDDs* can be pivotal for this purpose.

## Methods

### Database search and BLAST

A BLAST search was conducted using three different databases for tomatoes (Solgenomics network; https://solgenomics.net/, Plaza 5.0; https://bioinformatics.psb.ugent.be/plaza/ and Gramene; https://www.gramene.org. Arabidopsis and Rice sequences were verified using TAIR10 (https://www.arabidopsis.org/) and RAP-DB (https://rapdb.dna.affrc.go.jp/) respectively. Default parameters were used as the conditions for BLAST searches.

### Multiple alignment and phylogenetic tree construction

Multiple protein sequence alignments were performed using ClustalW and visualized using the ALIGNMENTVIEWER (https://github.com/sanderlab/alignmentviewer) software. A phylogenetic tree was constructed using MEGA (version 11.0; Penn State University, PA, USA) and the maximum likelihood tree method (bootstrap 1000 replicates). Sequences for the multiple alignments and phylogenetic tree and accession numbers of *OsIDD*s and *AtIDD*s are available in Table S12–S14. The iTOL web tool was used to construct the evolutionary tree (https://itol.embl.de).

### Chromosomal location, synteny analysis, motif visualization, and 3D structure visualization

The locations of candidate genes were acquired from the Solgenomics network (https://solgenomics.net/), and positions were visualized using MG2C v2.1 (http://mg2c.iask.in/mg2c_v2.1/). Synteny analysis and Ka/Ks values were calculated using TBtools^130^. Gene duplications were assessed by using R package “Doubletrouble” (https://github.com/almeidasilvaf/doubletrouble)^131^. The MEME suite was used to identify and visualize conserved motifs among candidate genes (https://meme-suite.org/meme/tools/meme). Motifs were searched among the given sequences, and the remainder were set to default. The 3D structure was identified using a UniProt (https://www.uniprot.org) database search and visualized using Afphafold2.0 (https://www.uniprot.org/database?query=(name:AlphaFoldDB)&direct).

### *Cis*-regulatory motif analysis and Coexpression network construction

Promoter sequences of 3 Kb of each *SlIDD* gene were used to scan and identify *cis*-regulatory elements using FIMO (https://meme-suite.org/meme/tools/fimo) against Arabidopsis promoter matrices (http://bar.utoronto.ca/~nprovart/ArabidopsisDAPv1.meme) based on previous reports^11,66,132,133^. The cutoff values for the p- and q-values were 1.99E-14 and 1.63E-09 respectively. TB tools were used to visualize the architectural positions of the major promoter elements^130^. A coexpression network was constructed using the TomExpress database^134^. STRING (https://string-db.org) was used to identify interaction partners of *SlIDDs.*

### Plant materials and growth conditions

All experiments were conducted using *Solanum lycopersicum* cv*.* M82 seeds kindly provided for the experiments by Prof. Soon Ju Park from Gyeongsang National University, Jinju, Korea. The plants were grown under long-day conditions and controlled temperatures in a greenhouse at Wonkwang University, Iksan, South Korea. Plants were grown under natural and supplemental light from a natrium, and halogen lamps were applied in the early morning and late evening. The light/dark cycle was 16 h/8 h/day. Plants were supplied with nutrients in the irrigation water one month after transplanting, following the manufacturer’s guidelines (S-feed, 1 kg/10 a/day; https://www.farmhannong.com/kor/product/product_ct01/view.do?seq=4392 (accessed on 08 November 2023).

### Abiotic stress treatment

Stress treatments were applied using potting media to ensure regular greenhouse growth. Salt stress was induced by saturating the potting medium with tap water mixed with 200 mM NaCl at an adjusted pH of 6.8. Proton stress was induced by saturating the plants with tap water at pH 4.2. Flood stress was induced by submerging plant roots in potting media in water at a pH of 6.8. All stress treatments were performed under greenhouse conditions. Control plants were saturated with water at pH 6.8. Shoot and root samples were collected at 2 and 24 h after treatment.

### RNA extraction and quantitative real time PCR for stress-responsive *SlIDDs*

To extract RNA from shoots and roots, 3 weeks old control and treated plants were harvested at 2 pm in a greenhouse. Total RNA was extracted using the AccuPrep® Universal RNA extraction kit (Bioneer, Daejeon, Korea) and treated with RNase-free DNase to remove DNA fragments (Qiagen, Hilden, Germany). One microgram of total RNA was used to synthesize cDNA with *AccuPower*® RT PreMix (Bioneer, Daejeon, Korea). qRT-PCR was performed using a T100TM Thermocycler system (Bio-Rad, Hercules, CA, USA). Primer information is provided in Table S11. Reactions (10 µL final volume) were prepared using 5 µL of LaboPass™ SYBR Green Q master kit (Cosmogenetech, Dajeon, Korea). Next, 0.5 pmol of a primer pair, and 0.5 µL of cDNA template. Four biological samples and two technical replicates were used for quantification. Ubiquitin was used as a reference. gene expression analysis was performed with the 2^ − ΔΔCt method using Bio-Rad CFX Maestro software v.4.0 (Bio-Rad). The baseline and threshold levels were set according to the manufacturer’s instructions.

### Ethics approval and consent to participate

All experiments were conducted in greenhouses situated at Wonkwang University using wild-type plants. Ethical guidelines provided by the ethics committee were followed when conducting the experiments.

### Supplementary Information

Supplementary Figure S1.

Supplementary Figure S2.

Supplementary Figure S3.

Supplementary Tables.

## Data Availability

All data related to the expression analyses are available in the GEO repository under accession number GSE248090 (https://www.ncbi.nlm.nih.gov/geo/query/acc.cgi?acc=GSE248090). Sequences related to the bioinformatics analyses are included in the Supplementary Tables.
