# Supplementary material for: Functional characterization of plant specific Indeterminate Domain (IDD) transcription factors in tomato (Solanum lycopersicum L.)
Source: Sci Rep. 2024 Apr 5;14:8015. doi: 10.1038/s41598-024-58903-0 (PMC10997639; doi:10.1038/s41598-024-58903-0)
Supplement: Supplementary file 1 — Supplementary Figure S1. [file 41598_2024_58903_MOESM1_ESM.pdf]

```
Solyc01g005060_SlIDD12      ............................................................
Solyc01g007120              ............................................................
Solyc01g099340_SlIDD10      ............................................................
Solyc02g062940_SlIDD17      ............................................................
Solyc02g085580_SlIDD1       ............................................................
Solyc03g098070              ............................................................
Solyc03g121660_SlIDD11      ............................................................
Solyc04g008500_SlIDD4       ............................................................
Solyc04g056320_SlIDDb       ...................................MSFTGDLRH.........LPPN...
Solyc04g080130_SlIDD18      ............................................................
Solyc05g054030_SlIDD5       ............................................................
Solyc06g062670_SlIDD2       ............................................................
Solyc06g065440              ..........................................................ME
Solyc06g072360_SlIDD9       ............................................................
Solyc06g075250_SlIDD7       ............................................................
Solyc07g053570_SlIDD8       ............................................................
Solyc08g063040_SlIDD16      ............................................................
Solyc08g080850              ..........................MEELADKIEIPQKAEDKKDEELEELEDEDEEGEL
Solyc09g007550_SlIDD13      ............................................................
Solyc09g065670_SlIDD15      ............................................................
Solyc09g074780_SlIDD3       ............................................................
Solyc10g084180_SlIDD14      ............................................................
Solyc11g017140              .....MPSDNHSFTNLN......................LHEQKWE..GSSYLDQEIRIE
Solyc11g066420              ......................................MIQGTSSSANNPQ.NIPLYS.S
Solyc11g069240_SlIDD6       ............................................................
AT1G03840_MGP/AtIDD3        ............................................................
AT1G14580_BLJ/AtIDD6        ............................................................
AT1G25250_FAL/AtIDD16       ............................................................
AT1G34370_AtSTOP1           ....METEDDLCNTNWGSSSSKSREPGSSDCGNSTFAGFTSQQKWE..DASILDYEMGVE
AT1G55110_AtIDD7            ............................................................
AT1G68130_AtIDD14           ............................................................
AT2G01940_SGR5/AtIDD15      ............................................................
AT2G02070_RVN/AtIDD5        ............................................................
AT2G02080_IME/AtIDD4        ............................................................
AT2G24990_AtIDDa            ..........................MEEVVLVTEPPPTIPP..VVEYEEEHDDDDDDDL
AT3G13810_AtIDD11           ............................................................
AT3G45260.1_BIB/AtIDD9      ............................................................
AT3G50700_AtIDD2            ............................................................
AT4G02670_AtIDD12           ............................................................
AT5G03150_JKD/AtIDD10       ............................................................
AT5G22890_STOP2             ..................................MHIHMMNRDEHIAKKVEGSISSFSGE
AT5G37350_AtIDDb            ..........................MEEVVVVAEPPPKITP..FVEYEEELEDDDDD.L
AT5G44160_NUC/AtIDD8        ............................................................
AT5G60470_EGRET/AtIDD13     ............................................................
AT5G66730_ENY/AtIDD1        ............................................................
Os03g0197800_OsIDD1         ............................................................
Os01g0195000_OsIDD2         ............................................................
Os09g0555700_OsIDD3         ............................................................
Os02g0672100_OsIDD4         ............................................................
Os07g0581366_OsIDD5         ............................................................
Os08g0554400_OsIDD6         ............................................................
Os02g0518500_OsIDD7         ............................................................
Os01g0242200_OsIDD8         ............................................................
Os01g0935000_OsIDD9         ............................................................
Os04g0566400_OsIDD10        ............................................................
Os01g0572300_OsIDD11        ............................................................
Os08g0467100_OsIDD12        ............................................................
Os09g0449400_OsIDD13        ............................................................
Os03g0237250_OsIDD14        ............................................................
Os01g0888700_OsIDDa         ............................................................
Os01g0871200_OsIDDb         MDSGLGRSSETSLKALPSMASNATRNTDPDQQGVRFSSMDQPPCFARPGQSFPAFPPLFG
Os03g0838800_OsIDDc         ....................................MYLEQDEDFSQMIMELCDFDASST
Os04g0165200_ART2           ......................................MNPQ.................E
Os07g0162300_OsIDDe         ..........................MKRSGRTPTWP.......................
Os10g0419200_Ehd2           ............................................................
Os08g0562300_OsIDDf         ......................................MIPGGGGGGGGGG..ISPYLVQ
Os02g0572900_STOP1          ....................................MKSTTHLLAGGGGGGSVAPPTAAT
Os12g0170400_DART1          .......MDRDQMTN.......................TMRDQAANLTSMNPLFYPFMAD
```

Figure S1. Multiple alignment of *IDD*-like transcription factors in Arabidopsis, Tomato and Rice. highly conserved amino acids are indicated in red characters and boxes.

```
                                                                        1
Solyc01g005060_SlIDD12   ...............................................MLDN.........
Solyc01g007120           ..................................MKLDVNVLRYLSKDDFRVLTAVEMGM
Solyc01g099340_SlIDD10   ...................................MSNSNLSSG..NSSE.....EADET
Solyc02g062940_SlIDD17   ....................................................MNDCVGSG
Solyc02g085580_SlIDD1    ..........................................MPADPDNSSAMNDSTGSG
Solyc03g098070           ............................................................
Solyc03g121660_SlIDD11   ............MKDLMNQQAQVPGVVMEENMSNLTSASG.EASVSSSNR.....NDNNN
Solyc04g008500_SlIDD4    ................................M..REE......ELSQ............
Solyc04g056320_SlIDDb    ......TIGD.......................DPMVPLLNLSAVQTRMDSLQKFLSDSV
Solyc04g080130_SlIDD18   ....................................................MAEIEKSA
Solyc05g054030_SlIDD5    .........................................MLTSNSFLFG........A
Solyc06g062670_SlIDD2    ...............................MSIVTCEEA.AASLSSSS........NMN
Solyc06g065440           QQAN..............QSNENHDF.AGLQEWDPRAMLKSMSFLEQKIHQLRELVRIIV
Solyc06g072360_SlIDD9    ..........................................................MT
Solyc06g075250_SlIDD7    ..................................MMSD..DGLSSLAFI..........Q
Solyc07g053570_SlIDD8    ..............................................MTEEV...ISN..G
Solyc08g063040_SlIDD16   ...........MIKGMLGDDSMSN..........LTSASNEASISSTNNR.....IEIGS
Solyc08g080850           SWSSDSEIGEALDYLDTKDNSESNVGAFSLQTRRPNAHGGLHTRPNSSALQPLSNRTQKF
Solyc09g007550_SlIDD13   ..................................MMSG..DLFSIHQ............Q
Solyc09g065670_SlIDD15   ............................................................
Solyc09g074780_SlIDD3    ...................................MSNISGEEGSFSSGE.....EAHLH
Solyc10g084180_SlIDD14   ..................................MMSG..DVFSFPSSV......KAVLP
Solyc11g017140           QQFTGFAKPKHTSEMD.QQGNQNHDT.TRIHDWDPRALLNNLSFLEQKIHQLQELVHLIV
Solyc11g066420           NHHPNNN........IQEDIFSSSSSSYQQN.....SFLFNLSLLKEKVHQVQSLATMFI
Solyc11g069240_SlIDD6    ..................................MMSN..DGLS................
AT1G03840_MGP/AtIDD3     ..............................................MTTED.QTISSSGG
AT1G14580_BLJ/AtIDD6     ..............MDLCSFSKNRP....AEKM..SSSYNTIALSSTPTFLLSSAAAGPG
AT1G25250_FAL/AtIDD16    .......................................MIHYEQNNNLQNL........
AT1G34370_AtSTOP1        PGLQES...........IQANVDFLQGVRAQAWDPRTMLSNLSFMEQKIHQLQDLVHLLV
AT1G55110_AtIDD7         .........MMMNRDILFHQQQQQ..QMEENMSNLTSASGDQASVSSGNR.....TETSG
AT1G68130_AtIDD14        .......................................MIDYERSNTTKNINTHHHNPP
AT2G01940_SGR5/AtIDD15   .........................................MRTDQVMLSNKNTNTCCVV
AT2G02070_RVN/AtIDD5     ...............MAASSSSAAS....FFGV..RQD......DQSHLLPPNSSAAAPP
AT2G02080_IME/AtIDD4     ................MSSSSYNTS....VIPS..SSS......SAQPFFITSS...GTG
AT2G24990_AtIDDa         SLSSDSDIAEALDWLDGKDDDELIGGGFSLHARRPNAHGGHGSRPNSSSLQPISNKAQKL
AT3G13810_AtIDD11        ...............MLLHQHQQP..QQDENMSNLTSASGDQASVSSGNI.....TEASG
AT3G45260.1_BIB/AtIDD9   .................................MMMPDDHHPLSFPSYV..........L
AT3G50700_AtIDD2         ..........................................MPVDLD.....NSSTVSG
AT4G02670_AtIDD12        .......................MDMFSSHSLSYKLSSLSTEASASSGNNTLS..TIQEF
AT5G03150_JKD/AtIDD10    ................................MQMIPG..DPFSISSSMGGFVHQETHLH
AT5G22890_STOP2          TSTSSKQ........IYVNPVTTTGTKSMEDDDVSLSLLYNLSTLHEKVHQIQSLVSFYM
AT5G37350_AtIDDb         SLSSDSDIAEALDWLDGKDDDELIGGGFSLHARRPNAHGGHGSRPNSSALQPISNKAQKL
AT5G44160_NUC/AtIDD8     ..............................................MTSEVLQTISSGSG
AT5G60470_EGRET/AtIDD13  .............................MNQDMMIPHNHDRLSFSSFV..........H
AT5G66730_ENY/AtIDD1     ..........................................MPVDLD.....NSSTVSG
Os03g0197800_OsIDD1      ..............................................MEVETSSGTAERGA
Os01g0195000_OsIDD2      ...........MMLKDLAEIQQQQ..........LLAAAEEN....MSN.......LTSA
Os09g0555700_OsIDD3      .................MAAASSAP....FFGL...GD.........TQMPPPQNPTNPA
Os02g0672100_OsIDD4      .............MASNSSAAAVAA....LFGI.RDGDHEDQIKPLFAQQQQHHHHQPPM
Os07g0581366_OsIDD5      ...............................................GGWSCGGGRSRGD
Os08g0554400_OsIDD6      .................MAAASSAH....YFGL...GE.........PQMQ..QQQQQPP
Os02g0518500_OsIDD7      ...................................MSHTSEEESLNNLQQ.....QPKLE
Os01g0242200_OsIDD8      ..............................................MATSEAAAISNPFA
Os01g0935000_OsIDD9      ...............MEEFELQQQPYYSK..............LLLGSPLEIANVDDSDL
Os04g0566400_OsIDD10     .............MASNSSAAAAAA....FFGISRDGDQHDQIKPLISHQQHQHQQQQLA
Os01g0572300_OsIDD11     ...........MFHHHLQQQQQQEE..EEGGGLFLVEEAAEA....ADQQ.....QESSM
Os08g0467100_OsIDD12     ........................................MLSSCAPTAVPPQDASGAAT
Os09g0449400_OsIDD13     ........................................MLGSCAPTTAGPPPDEATTP
Os03g0237250_OsIDD14     ..............................MALVKSHHQMLASSSTSSSSPSSQQQQPPP
Os01g0888700_OsIDDa      ..................................MKLDVNALRYLSKDDFRVLTAVEMGM
Os01g0871200_OsIDDb      VQSSSLYLPDDIEAKIGNQFESNPSPNNPTMDWDPQAMLSNLSFLEQKIKQVKDIVQSMS
Os03g0838800_OsIDDc      TQAR..................HGGEAAAAAAGNARAVLTYLTFLEQKIGHLRGIISSTP
Os04g0165200_ART2        FQFFQGV........EQEMLLLSHGGGGAAAAASDDVLAYHVGVLRDKVQQLEPLVGMVV
Os07g0162300_OsIDDe      MRSTGS........................TARRPNAHGGVLSRP....FQPISNRTQKL
Os10g0419200_Ehd2        ..............MLLSDLSSDQEATGSNSHGGGGGDRMVVGSHGAAHVVLSNLFLPPA
Os08g0562300_OsIDDf      SQHGHGG........GVDGMEMEEGGGFMGEQPQCHPLLYNLSVLKDRVQQLHPLVGLAV
Os02g0572900_STOP1       APTLAMEVGDP....SGALVGGAGGAGAGGGDGDARDALLRLVALGDRMAAVRRRIAASI
Os12g0170400_DART1       DALLGMAPP........PPQQLLPSVSIQHMDWSPDTMLDNLTFIEEKIRQVKDVIRSMA
```

Figure S1. Multiple alignment of *IDD*-like transcription factors in Arabidopsis, Tomato and Rice. highly conserved amino acids are indicated in red characters and boxes.

```
                                  10                  20
Solyc01g005060_SlIDD12   .SSSS...........EALINSGD.........SNRRKR....RPAGT............
Solyc01g007120           RNHEIVP..CELIDRIARLKHGGTYKVLKNLLKHKLVHHDSSKYDGFR............
Solyc01g099340_SlIDD10   P..YV.........LS..STSDGSSAHQQHSQTNNKKRR....KLPGN............
Solyc02g062940_SlIDD17   EVSVL..........SSGDQTAPCKE.......AAKKKR....NLPGM............
Solyc02g085580_SlIDD1    EASVS..........SSGNQVVPLKE.......SAKKKR....NLPGM............
Solyc03g098070           ............................................................
Solyc03g121660_SlIDD11   NNNNNIYPHHQYNFAPPNQQTQPAQQ........IKKKR....NQPGN............
Solyc04g008500_SlIDD4    ....N.........QQENLLQAQ........APS.KKRR....NQPGT............
Solyc04g056320_SlIDDb    NSNT.......LLGQHQMNMVSDEITSAIHQIIVNGAALLSSAQLTNP............
Solyc04g080130_SlIDD18   EESII.............QQNFP...........VKRKR....NLPGN............
Solyc05g054030_SlIDD5    PSNYS...........DPFSSPENG.......FIIKRKR....RPAGT............
Solyc06g062670_SlIDD2    NDTNG.......AFCYPPQHQLVTQQ........IKKKR....NQPGN............
Solyc06g065440           DHRSLAGIQGSDLSIQQQQLITADLTSIIIQLISTAGSLLPTVKHQAN...PPTKRLEQ.
Solyc06g072360_SlIDD9    SSEIN...........ENSCINNNN........NNKRKR....RPAGT............
Solyc06g075250_SlIDD7    DPNSN.........PTNNNPNPNSN.......PSAKRKR....NLPGK............
Solyc07g053570_SlIDD8    FTHDN.........SIE............GSNPPPLKKK...RNLPGN............
Solyc08g063040_SlIDD16   HVLYP.........QLQNIQTQPPP.........NKKKR....NLPGN............
Solyc08g080850           ANHIRASPLEEWEGRIKVGMSNSVTTAIRGSVRDMAIGKTKTTEKADR............
Solyc09g007550_SlIDD13   QHQQV.........LVEQNPNPKAN.......SSSKKRR....NLPGT............
Solyc09g065670_SlIDD15   .............MVSNHNHHEQPPK........IKKKR....NLPGN............
Solyc09g074780_SlIDD3    TSNNN.........ISSITTSNGSTSQSQQQQPLAKKKR....NLPGN............
Solyc10g084180_SlIDD14   HHHQE.........LQTANPNPKPN.......TSSKKRR....NLPGT............
Solyc11g017140           GRRGQDEVQGNDLIVQQQQLITADLTSIIVQLISTAGSLLPTMKYTLSSAIPAASQLGQ.
Solyc11g066420           TPDNQT...IIHPPPESISMIIANMGTLIQEIITTSSSLMFSCQKIVLDSTSLSQNSSRY
Solyc11g069240_SlIDD6    .................................CSMR.......................
AT1G03840_MGP/AtIDD3     YVQSS.........STTDHVDHHHHDQHESLNPPLVKKK...RNLPGN............
AT1G14580_BLJ/AtIDD6     PNNFN.........RQEAAMTMVQQQPTSSVAPPPKKRR....NQPGN............
AT1G25250_FAL/AtIDD16    PSSSS...........NDLLLGING.....ADATHKRKR....RPAGT............
AT1G34370_AtSTOP1        GRGGQLQGRQDELAAQQQQLITTDLTSIIIQLISTAGSLLPSVKHNMS..TAPGPFTGQ.
AT1G55110_AtIDD7         SNINQ.........HHQEQCFVPQSS........LKRKR....NQPGN............
AT1G68130_AtIDD14        PSSSS...........SDLLPDGNG.....TAVTQKRKR....RPAGT............
AT2G01940_SGR5/AtIDD15   SSSSS...........DPFLSSSENGVTTTNTSTQKRKR....RPAGT............
AT2G02070_RVN/AtIDD5     PPPPH.........HQAPLPPLE........APPQKKKR....NQPRT............
AT2G02080_IME/AtIDD4     DNDFN.........RKDTFMSMIQQPNSS..APPPKKRR....NQPGN............
AT2G24990_AtIDDa         TSHVRASPLEGWEGRVKVGMSNSVTTAIRGSLRDTEIGRSRNTDKADR............
AT3G13810_AtIDD11        SNYFP.........HHQQQQEQQQQQ........IQKLS....CSWTDSLFQLFDTVTFL
AT3G45260.1_BIB/AtIDD9   HQEHI.........APNPNPNPNPTSS.....NSAKRKR....NLPGN............
AT3G50700_AtIDD2         EASVSI........SSTGNQNPLPNS.......TGKKKR....NLPGM............
AT4G02670_AtIDD12        SGFHN........VISSVCTHTETHK........PKKKR....GLPGN............
AT5G03150_JKD/AtIDD10    HLQQQ.........IPDLNPNSNPNPNAKPNSSSAKKKR....NQPGT............
AT5G22890_STOP2          VSTNN.....INQSSGSTSLAVANIGSLVQEIITAASSMLYTCQQLQIGSN.........
AT5G37350_AtIDDb         TSHVRASPLEGWEGRVKVGMSNSVTTAIRGSLRDTEIGRSRNTDKADR............
AT5G44160_NUC/AtIDD8     FAQPQ.........SSS.TLDHDES....LINPPLVKKK...RNLPGN............
AT5G60470_EGRET/AtIDD13  DQEHI.........TPNPYPNSQPAAST....KTPKKKR....NLPGN............
AT5G66730_ENY/AtIDD1     DASV..........SSTGNQNLTPKS.......VGKKKR....NLPGM............
Os03g0197800_OsIDD1      GAGAQ..........QQPPPQPQPQP.......PAKKKR....ALPGM............
Os01g0195000_OsIDD2      SGDQA.........SVSSHPAPPP..........AKKKR....SLPGN............
Os09g0555700_OsIDD3      LHHHP.........NPSPAPVAA.......AAPAPKKKR....NQPGN............
Os02g0672100_OsIDD4      APSNA.........AAAASAAGSAAGQAAVAAPPAKKKR....TLP..............
Os07g0581366_OsIDD5      .ATQS..........DGDGGGARAGG.......GAGSGG....GGGGA............
Os08g0554400_OsIDD6      LQNN............AAAPVAA.......TPP.PKKKR....NQPGN............
Os02g0518500_OsIDD7      A...................CAAGSSKGDTVMPVVKKRR....GHPGN............
Os01g0242200_OsIDD8      PLTNH.........QQE..........HPPPPPPPAKKK...RNLPGT............
Os01g0935000_OsIDD9      QLVAG.........VPS..........DPPPAPPTAVKKKKKRSLPGT............
Os04g0566400_OsIDD10     ASLTG.........VATAAPTAASSQGAPPAAPPAKKKR....NLPA.............
Os01g0572300_OsIDD11     SNLTS.........SASTVPPPPPPSSGNNGNNSNKRKR....SLPGN............
Os08g0467100_OsIDD12     EPFRS...........LQIATAGA.........AAKKKR....RPAGT............
Os09g0449400_OsIDD13     EPFRS...........LQIATASAG........SAKKKR....RPAGT............
Os03g0237250_OsIDD14     PASNS...........SSLAAAAAD.....QPSPAKRKR....RPPGT............
Os01g0888700_OsIDDa      RNHEIVP..AELVDRIAGLKHGGTYKVLRNLLKNKLVHHDATKYDGYR............
Os01g0871200_OsIDDb      NRESQVAG.GSSEAQAKQQLVTADLTCIIIQLISTAGSLLPSMKNPISS.NPALRHLSNT
Os03g0838800_OsIDDc      N..............PPPQIVAAELSCIVVQLVSISKNLAAARR................
Os04g0165200_ART2        VSPAAP...HGQLRRDAAAMAASSACSVLQEITAAASAVAHRLEQANAASS.......
Os07g0162300_OsIDDe      ASHIRATPLEEWEGRMNVGMSNSVTTAIRDSIRETAIGKTRNTEKADR............
Os10g0419200_Ehd2        AAAAAT......MLLPAAPVMVRPAAMAAAQEPRAKKKR....SLPGN............
Os08g0562300_OsIDDf      AHNAHA...HGPLD.......VSAADAIIQEIVAAASSMMYAFQLLCDLGT.........
Os02g0572900_STOP1       SGESG......PLSSSDIHSVSSEISSAAHLVVLNAASLLSSTVASLP............
Os12g0170400_DART1       GRRAS....SSSAATPEQQLVNADLTCLIVQLISTAGSLLPSLKNSSFLSRTTPPPAAA.
```

Figure S1. Multiple alignment of *IDD*-like transcription factors in Arabidopsis, Tomato and Rice. highly conserved amino acids are indicated in red characters and boxes.

```
Solyc01g005060_SlIDD12      ............................................................
Solyc01g007120              ............................................................
Solyc01g099340_SlIDD10      ............................................................
Solyc02g062940_SlIDD17      ............................................................
Solyc02g085580_SlIDD1       ............................................................
Solyc03g098070              ............................................................
Solyc03g121660_SlIDD11      ............................................................
Solyc04g008500_SlIDD4       ............................................................
Solyc04g056320_SlIDDb       ...................................PPPPPPPPRPSSSAELKINLKSNHK
Solyc04g080130_SlIDD18      ............................................................
Solyc05g054030_SlIDD5       ............................................................
Solyc06g062670_SlIDD2       ............................................................
Solyc06g065440              ......FGG...........ASVPSETGTN.IGALTCNGYVPKAK.DQLSHVDQMGDC..
Solyc06g072360_SlIDD9       ............................................................
Solyc06g075250_SlIDD7       ............................................................
Solyc07g053570_SlIDD8       ............................................................
Solyc08g063040_SlIDD16      ............................................................
Solyc08g080850              ............................................................
Solyc09g007550_SlIDD13      ............................................................
Solyc09g065670_SlIDD15      ............................................................
Solyc09g074780_SlIDD3       ............................................................
Solyc10g084180_SlIDD14      ............................................................
Solyc11g017140              ......VGG...........VTVPSTAGTS.AGGLTCNDGVAKLE.DQSNHIDQLRDCGI
Solyc11g066420              ....REPSQNDVGHGQGQGQVDHLLQDYDWYVDNYNSNCNTHEDNKNHVTSSSTIIASST
Solyc11g069240_SlIDD6       ............................................................
AT1G03840_MGP/AtIDD3        ............................................................
AT1G14580_BLJ/AtIDD6        ............................................................
AT1G25250_FAL/AtIDD16       ............................................................
AT1G34370_AtSTOP1           ......PGS...........AVFPYVREANNVASQSQNNNNCGAR.EFDLPKPVLVDE..
AT1G55110_AtIDD7            ............................................................
AT1G68130_AtIDD14           ............................................................
AT2G01940_SGR5/AtIDD15      ............................................................
AT2G02070_RVN/AtIDD5        ............................................................
AT2G02080_IME/AtIDD4        ............................................................
AT2G24990_AtIDDa            ............................................................
AT3G13810_AtIDD11           E...........................................................
AT3G45260.1_BIB/AtIDD9      ............................................................
AT3G50700_AtIDD2            ............................................................
AT4G02670_AtIDD12           ............................................................
AT5G03150_JKD/AtIDD10       ............................................................
AT5G22890_STOP2             ......NNNNDIDNDQ...TVDAMVLEFSRQETDPGHDFVQESTNLFGVQERGQISFP.D
AT5G37350_AtIDDb            ............................................................
AT5G44160_NUC/AtIDD8        ............................................................
AT5G60470_EGRET/AtIDD13     ............................................................
AT5G66730_ENY/AtIDD1        ............................................................
Os03g0197800_OsIDD1         ............................................................
Os01g0195000_OsIDD2         ............................................................
Os09g0555700_OsIDD3         ............................................................
Os02g0672100_OsIDD4         ............................................................
Os07g0581366_OsIDD5         ............................................................
Os08g0554400_OsIDD6         ............................................................
Os02g0518500_OsIDD7         ............................................................
Os01g0242200_OsIDD8         ............................................................
Os01g0935000_OsIDD9         ............................................................
Os04g0566400_OsIDD10        ............................................................
Os01g0572300_OsIDD11        ............................................................
Os08g0467100_OsIDD12        ............................................................
Os09g0449400_OsIDD13        ............................................................
Os03g0237250_OsIDD14        ............................................................
Os01g0888700_OsIDDa         ............................................................
Os01g0871200_OsIDDb         LCAPMILGTNCNLRPSANDEATIPDISKTHDYEELMNSLNTTQAESDEMMNCQNPCGGEG
Os03g0838800_OsIDDc         .......GG............................DDADADAKHDGS...........
Os04g0165200_ART2           .....ASFRRGVAFADDAHAAGGVQMFAAGAHGGGQGGDDEAGASLFHHHGPPAVGAPTV
Os07g0162300_OsIDDe         ............................................................
Os10g0419200_Ehd2           ............................................................
Os08g0562300_OsIDDf         .....APTT..APSQETAAASAVVVKNNDHAADAGQMEDDHLMQQQWQQNGSRQHDYSSH
Os02g0572900_STOP1          ...................................APPPPP...ATTVQELPPVVVS...
Os12g0170400_DART1          ......AGA..........AQAVSLAAGESSSSARNNETNREDEEEQMGSPDYDELFKVW
```

Figure S1. Multiple alignment of *IDD*-like transcription factors in Arabidopsis, Tomato and Rice. highly conserved amino acids are indicated in red characters and boxes.

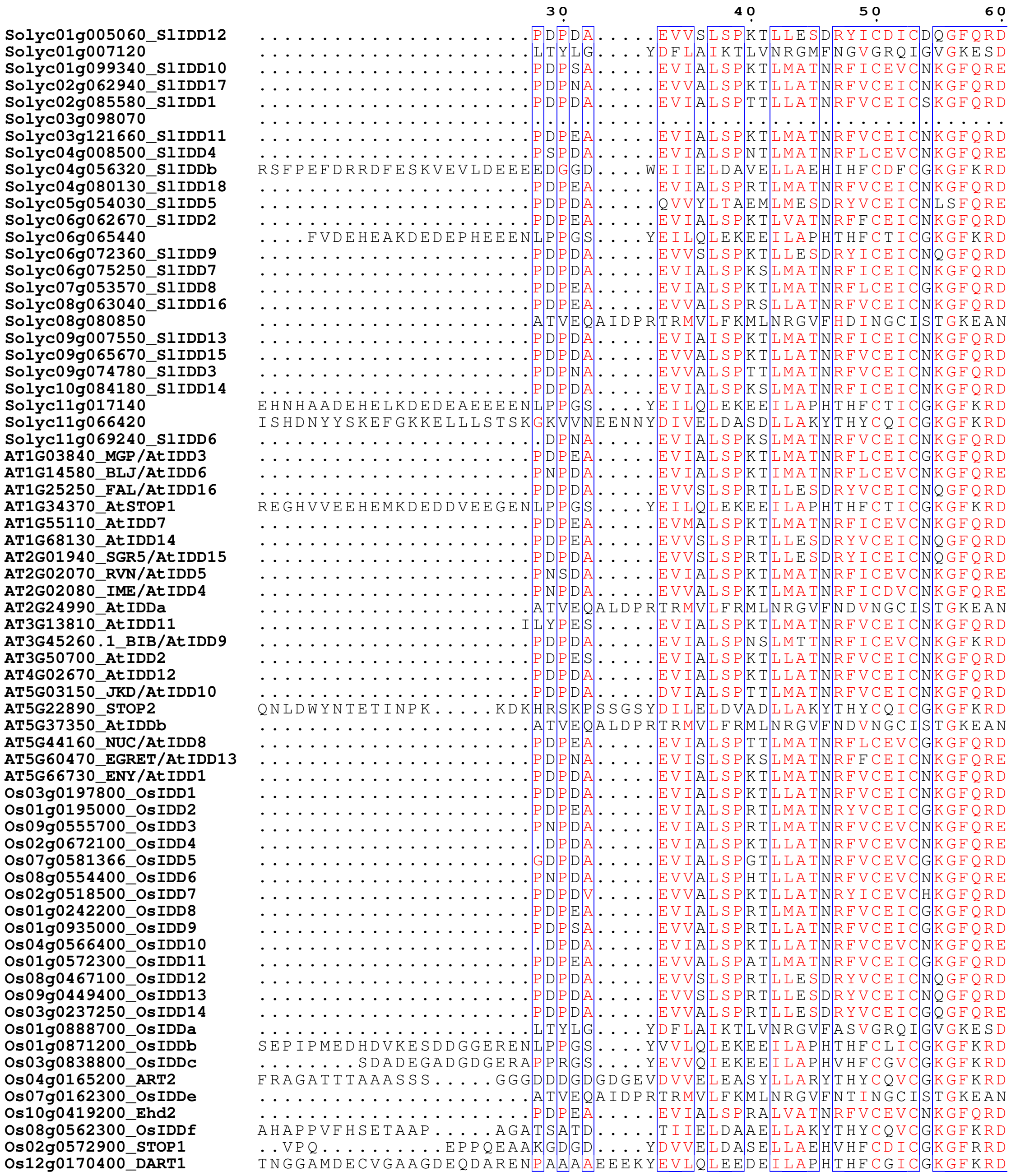

Figure S1. Multiple alignment of *IDD*-like transcription factors in Arabidopsis, Tomato and Rice. highly conserved amino acids are indicated in red characters and boxes.

```
                                     70        80                                90
Solyc01g005060_SlIDD12      QNLQMHRRRHKVPWKLVKR..E......................IEVKKRVFVCPEPSCL
Solyc01g007120              IFEVVKEDGTVLAMKLHRLG......................RVSFR....AVKSKRDYL
Solyc01g099340_SlIDD10      QNLQLHRRGHNLPWKLKQKT......................S.NEIKKRVYICPESSCI
Solyc02g062940_SlIDD17      QNLQLHRRGHNLPWKLRQRS.......................SNEVRKRVYYCPETTCV
Solyc02g085580_SlIDD1       QNLQLHRRGHNLPWKLRQRS.......................SNEVKKRVYYCPESSCV
Solyc03g098070              ....MHRRRHKVPWKLLKRET.......................PIVRKRVFVCPEPTCL
Solyc03g121660_SlIDD11      QNLQLHRRGHNLPWKLKQRT......................SKEIR.KKVYVCPETSCV
Solyc04g008500_SlIDD4       QNLQLHRRGHNLPWKLKQKN......................TKEVAKRKVYLCPEPTCV
Solyc04g056320_SlIDDb       ANLRMHMRAHGNQYKTPEALAKPEK..............CIDSSNSNKRRRFSCPFIGCT
Solyc04g080130_SlIDD18      QNLQLHRRGHNLPWKLRQRT.......................SNEVKKRVYVCPESSCV
Solyc05g054030_SlIDD5       QNLQMHRRRHKVPWKLKKKEEEKNE.................MDQVIKKRVYVCPEPSCV
Solyc06g062670_SlIDD2       QNLQLHRRGHNLPWKLKKRE......................NKEVVRKKVYICPESSCV
Solyc06g065440              ANLRMHMRGHGDEYKTSAALIKP...........QKD..SSSGTTLIKR..YSCPCIGCK
Solyc06g072360_SlIDD9       QNLQMHRRRHKVPWKLLKRET.......................PIVKKRVFVCPEPTCL
Solyc06g075250_SlIDD7       QNLQLHRRGHNLPWKLKQRN......................KNEVIKKKVYICPEKTCI
Solyc07g053570_SlIDD8       QNLQLHRRGHNLPWKLKQRT......................SKEV.RKRVYYCPEKVCV
Solyc08g063040_SlIDD16      QNLQLHRRGHNLPWKLKQRS......................NKE.VKKKVYVCPEASCV
Solyc08g080850              VYHATKADGQELAIKVYKTS......................VLVFKDRDRYVQGDYRFR
Solyc09g007550_SlIDD13      QNLQLHRRGHNLPWKLKQRN......................KQEIVKKKVYICPEKSCV
Solyc09g065670_SlIDD15      QNLQLHRRGHNLPWKLKQRT......................NKEN.KKKAYVCPEPTCV
Solyc09g074780_SlIDD3       QNLQLHRRGHNLPWKLRQRT......................T.AEVKKRVYICPEPTCV
Solyc10g084180_SlIDD14      QNLQLHRRGHNLPWKLKQRN......................KLEQVKKKVYICPEKTCI
Solyc11g017140              ANLRMHMRGHGDEYKTPAALAKP...........HKE..PSSEPTLIKR..YSCPYVGCK
Solyc11g066420              ANLRMHMRAHGDEYKSSAALSNPMKRI.......NDLTSDGSLKSSSNTIKYSCPQEGCR
Solyc11g069240_SlIDD6       QNLQLHRRGHNLPWKLKQRN......................NKEVIKKKVYICPEKSCV
AT1G03840_MGP/AtIDD3        QNLQLHRRGHNLPWKLKQRT......................SKEV.RKRVYVCPEKSCV
AT1G14580_BLJ/AtIDD6        QNLQLHRRGHNLPWKLKQKS......................NKEV.RRKVYLCPEPSCV
AT1G25250_FAL/AtIDD16       QNLQMHRRRHKVPWKLLKRDKKD.....................EEVRKRVYYCPEPTCL
AT1G34370_AtSTOP1           ANLRMHMRGHGDEYKTAAALAKP...........NKESVPGSEPMLIKR..YSCPFLGCK
AT1G55110_AtIDD7            QNLQLHKRGHNLPWKLKQRS......................NKDVVRKKVYYCPEPGCV
AT1G68130_AtIDD14           QNLQMHRRRHKVPWKLLKRETN......................EEVRKRVYYCPEPTCL
AT2G01940_SGR5/AtIDD15      QNLQMHRRRHKVPWKLLKRDNN......................IEVKKRVYYCPEPTCL
AT2G02070_RVN/AtIDD5        QNLQLHRRGHNLPWKLKQKS......................TKEV.KRKVYLCPEPSCV
AT2G02080_IME/AtIDD4        QNLQLHRRGHNLPWKLKQKS......................TKEV.KRKVYLCPEPTCV
AT2G24990_AtIDDa            VYHATKSDGSELAIKVYKTS......................VLVFKDRDRYVQGDYRFR
AT3G13810_AtIDD11           QNLQLHRRGHNLPWKLKQRS......................NKEVIRKKVYYCPEASCV
AT3G45260.1_BIB/AtIDD9      QNLQLHRRGHNLPWKLKQRT......................NKEQVKKKVYICPEKTCV
AT3G50700_AtIDD2            QNLQLHRRGHNLPWKLRQKS.......................NKEVKKKVYYCPEVSCV
AT4G02670_AtIDD12           QNLQLHRRGHNLPWKLKQKN......................TKEQQKKKVYYCPETNCA
AT5G03150_JKD/AtIDD10       QNLQLHRRGHNLPWKLKQRS......................KQEVIKKKVYICPIKTCV
AT5G22890_STOP2             ANLRMHMRAHGDEYKTREALISPTS.........QDKKGGYSLKKHY....YSCPQHGCR
AT5G37350_AtIDDb            VYHATKSDGSELAIKVYKTS......................VLVFKDRDRYVQGDYRFR
AT5G44160_NUC/AtIDD8        QNLQLHRRGHNLPWKLKQRT......................SKEV.RKRVYVCPEKTCV
AT5G60470_EGRET/AtIDD13     QNLQLHKRGHNLPWKLKQKT......................NKNQVKKKVYICPEKSCV
AT5G66730_ENY/AtIDD1        QNLQLHRRGHNLPWKLRQRS.......................TKEVRKKVYYCPVSGCV
Os03g0197800_OsIDD1         QNLQLHRRGHNLPWKLRQRS.......................GKEVRKRVYYCPEPTCV
Os01g0195000_OsIDD2         QNLQLHRRGHNLPWKLKQRN......................PKEVVRKKVYYCPEAGCV
Os09g0555700_OsIDD3         QNLQLHRRGHNLPWKLKQKN......................PKEA.RRRVYLCPEPSCV
Os02g0672100_OsIDD4         QNLQLHRRGHNLPWKLKQKN......................PLQAQRRRVYLCPEPTCV
Os07g0581366_OsIDD5         QNLQLHRRGHNLPWRLRQRGP.....................GAAPPRRRVYYCPEPGCV
Os08g0554400_OsIDD6         QNLQLHRRGHNLPWKLKQKN......................PKET.RRRVYLCPEPSCV
Os02g0518500_OsIDD7         QNLQLHRRGHNLPWKLKQRS......................S.TEAKKKVYYCPEITCP
Os01g0242200_OsIDD8         QNLQLHRRGHNLPWKLRQRS......................GKEP.RKRVYYCPEKSCV
Os01g0935000_OsIDD9         QNLQLHRRGHNLPWKLRQRGGAG.GGGG..............GEPP.RKRVYYCPEASCV
Os04g0566400_OsIDD10        QNLQLHRRGHNLPWKLKQKN......................PAQAQRRRVYLCPEPTCV
Os01g0572300_OsIDD11        QNLQLHRRGHNLPWKLKQRG......................SKEAVRKKVYICPEASCV
Os08g0467100_OsIDD12        QNLQMHRRRHKVPWKLLKREAG......................EAARKRVFVCPEPTCL
Os09g0449400_OsIDD13        QNLQMHRRRHKVPWKLLKREAG......................EAARKRVFVCPEPTCL
Os03g0237250_OsIDD14        QNLQMHRRRHKVPWRLVKRPAAATAAEDGGAAGGGGGAGGGAGGGGARKRVFVCPEPSCL
Os01g0888700_OsIDDa         IFEVATEDGTVLAMKLHRLG......................RTSFR....AVKSKRDYL
Os01g0871200_OsIDDb         ANLRMHMRGHGDEYKTAAALAKP...........SKD..SSLESAPVTR..YSCPYVGCK
Os03g0838800_OsIDDc         ANLRMHMRGHGEEYKSAAALAKPGG.........SPSRSPAAADAAARRRFYSCPYVGCK
Os04g0165200_ART2           ANLRMHMRAHGDEYKTAAALT................STGAGMRAAARRCSYSCPAEGCR
Os07g0162300_OsIDDe         VYHASKADGQELAIKVYKTS......................VLVFKDRDRYVHGDYRFR
Os10g0419200_Ehd2           QNLQLHRRGHNLPWKLRHRAAAVSAVTT..............AAPAPRKRVYYCPEPTCV
Os08g0562300_OsIDDf         ANLRMHMRAHGDEYKSKAALSNPTK..........LLAKGGDETMAAAARKYSCPQEGCR
Os02g0572900_STOP1          ANLRMHMRAHGDRFKTLDALSRPGQ..............PKPPAG..REVRFSCPYAGCN
Os12g0170400_DART1          ANLRMHMRGHGDEYKSAAALAKPPP.........PPEG.EEQPPQPERR..YSCPHAGCK
```

Figure S1. Multiple alignment of *IDD*-like transcription factors in Arabidopsis, Tomato and Rice. highly conserved amino acids are indicated in red characters and boxes.

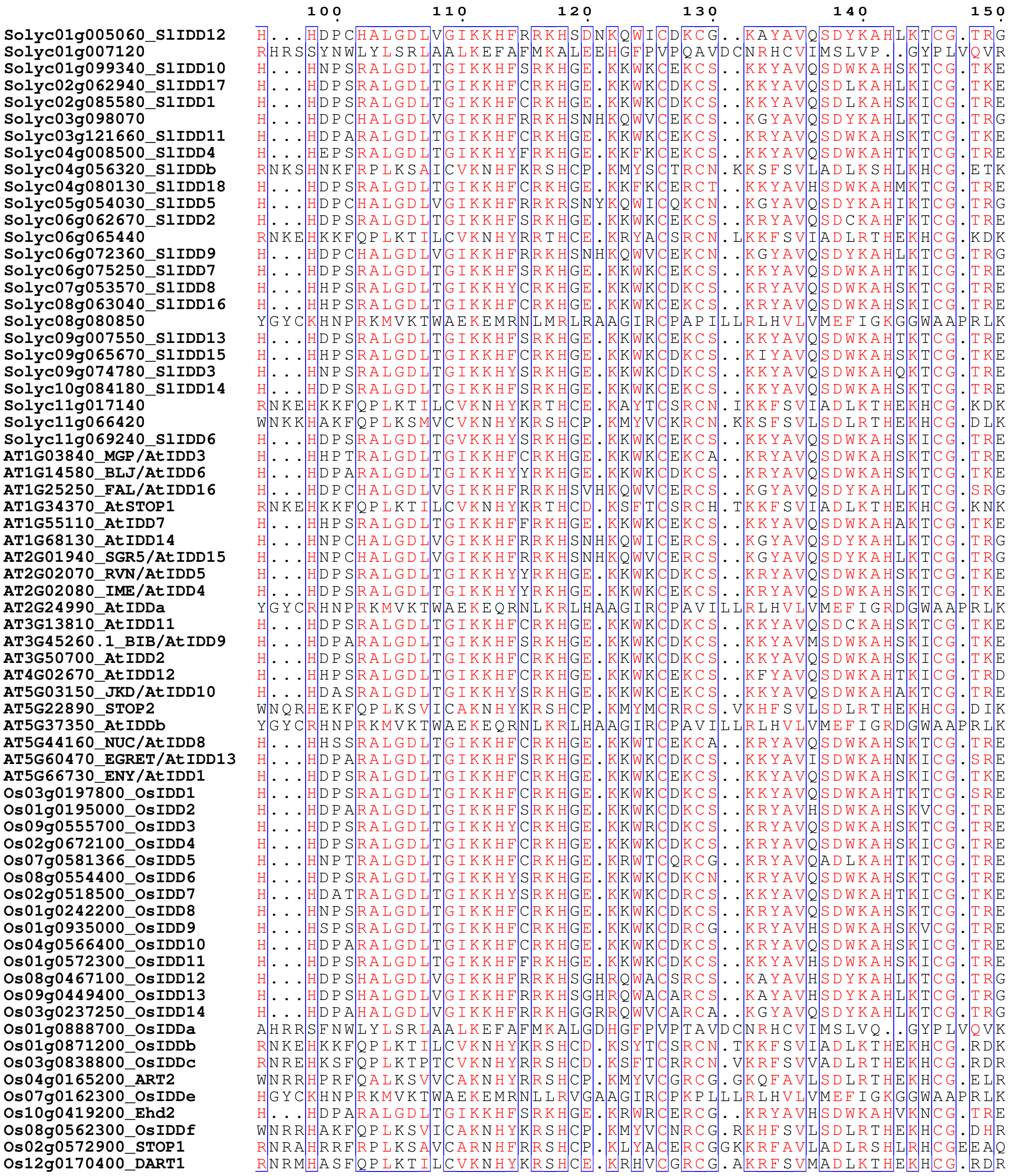

Figure S1. Multiple alignment of *IDD*-like transcription factors in Arabidopsis, Tomato and Rice. highly conserved amino acids are indicated in red characters and boxes.

```
                                 160         170
Solyc01g005060_SlIDD12    HSCDCGRVFS..RVESFIEHQDSCTIRRIR..............................
Solyc01g007120            ELQNP.....DVVFETIIAAVIRLAEHGLIHCDFNEFNIMIDDDE...............
Solyc01g099340_SlIDD10    YKCDCGTIFS..RRDSFVTHRAFCDALAEENNKVNQVLAS....................
Solyc02g062940_SlIDD17    YKCDCGTMFS..RRDSFITHRAFCDVLAKDTEKAQNAVTTPI..................
Solyc02g085580_SlIDD1     YKCDCGTLFS..RRDSFITHRAFCDALAQESAKTLPEKPPST..................
Solyc03g098070            HSCDCGRVFS..RVESFIEHQDACSMGR................................
Solyc03g121660_SlIDD11    YRCDCGTLFS..RRDSFITHRAFCDALAEESARAIT........................
Solyc04g008500_SlIDD4     YRCDCGTLFS..RRDSFITHRAFCDALVQENARN.ILSPNS...................
Solyc04g056320_SlIDDb     WKCSCGTSFS..RKDKLFGHMALFEGHMPAVET.......A...................
Solyc04g080130_SlIDD18    YRCDCGTLFS..RRDSFITHRAFCDALAQESARSHQPLMEDT..................
Solyc05g054030_SlIDD5     HSCDCGRVFS..RVETFIEHQDSCKPQSTTT.............................
Solyc06g062670_SlIDD2     YKCECGTIFS..RRDSFITHRAFCETLAMESARSVINE......................
Solyc06g065440            WLCSCGTTFS..RKDKLFGHISLFQGHTPAIPLDETKGFAQ...................
Solyc06g072360_SlIDD9     HSCDCGRVFS..RVESFIEHQDACSFGR................................
Solyc06g075250_SlIDD7     YKCDCGTLFS..RKDSFITHRAFCDALAEESARFTSVP......................
Solyc07g053570_SlIDD8     YRCDCGTIFS..RRDSFVTHRAFCDALAEETARVTAASNMH...................
Solyc08g063040_SlIDD16    YRCDCGTLFS..RRDSFITHRAFCDALTEESSRTIIP.......................
Solyc08g080850            DAALSSDKLRECYVEIIMAMRTLYQKCKLVHGDLSEYNILYFEG................
Solyc09g007550_SlIDD13    YKCDCGTLFS..RKDSFITHRAFCDALAEESARIT.........................
Solyc09g065670_SlIDD15    YRCDCGTLFA..RKDSFVTHRAFCDALAEESARLSANSG.....................
Solyc09g074780_SlIDD3     YKCDCGTIFS..RRDSFITHRAFCDALAEENNKVNQGLMNNN..................
Solyc10g084180_SlIDD14    YKCDCGTLFS..RKDSFITHRAFCDALAEESSRITSVG......................
Solyc11g017140            WLCSCGTTFS..RKDKLFGHISLFQGHTPAVSPDETKGSAG...................
Solyc11g066420            WQCSCGTTFS..RKDKLMGHVSLFVGHTPLI.............................
Solyc11g069240_SlIDD6     YKCDCGTLFS..RKDSFITHRAFCDALAEESTRFTSIQ......................
AT1G03840_MGP/AtIDD3      YRCDCGTIFS..RRDSFITHRAFCDALAEETARLNAASHLK...................
AT1G14580_BLJ/AtIDD6      YRCDCGTIFS..RRDSYITHRAFCDALIQESARNPTVSFTA...................
AT1G25250_FAL/AtIDD16     HSCDCGRVFS..RVESFIEHQDTCTIRQPQP.............................
AT1G34370_AtSTOP1         WLCSCGTTFS..RKDKLFGHIALFQGHTPAIPLEETKPSAS...................
AT1G55110_AtIDD7          YKCDCGTLFS..RRDSFITHRAFCDALAEESAR...........................
AT1G68130_AtIDD14         HSCDCGRVFS..RVESFIEHQDTCTVRRSQP.............................
AT2G01940_SGR5/AtIDD15    HSCDCG.FFSSFRVESFIEHQDNCSARRVHR.............................
AT2G02070_RVN/AtIDD5      YRCDCGTLFS..RRDSFITHRAFCDALAQESARH.PTSLTS...................
AT2G02080_IME/AtIDD4      YRCDCGTIFS..RRDSYITHRAFCDALIQETARNPTVSFTS...................
AT2G24990_AtIDDa          DAALSLDKLRECYLELIIQMRVLYQKCKLVHGDLSEYNILYFEG................
AT3G13810_AtIDD11         YRCDCGTLFS..RRDSFITHRAFCEALAEETAREVVIP......................
AT3G45260.1_BIB/AtIDD9    YRCDCGTLFS..RKDSFITHRAFCDALAEESARFVSVPP.....................
AT3G50700_AtIDD2          YKCDCGTLFS..RRDSFITHRAFCDALAEENARSHHSQSKKQ..................
AT4G02670_AtIDD12         YRCDCGTLFS..RKDTFITHRAFCDALAEESARLHSTS......................
AT5G03150_JKD/AtIDD10     YKCDCGTLFS..RKDSFITHRAFCDALTEEGARMSSLSNNNPV.................
AT5G22890_STOP2           WVCSCGTKFS..RKDKLMSHVSLFLGHVPAH.............................
AT5G37350_AtIDDb          DAALSLDKLRECYLELIIQMRVLYQKCKLVHGDLSEYNILYFEG................
AT5G44160_NUC/AtIDD8      YRCDCGTIFS..RRDSFITHRAFCDALAEETAKINAVSHLN...................
AT5G60470_EGRET/AtIDD13   FRCDCGTLFS..RKDSFISHRSFCDVLAEESSKFFSVPS.....................
AT5G66730_ENY/AtIDD1      YKCDCGTLFS..RRDSFITHRAFCDALAEESAKN.HTQSKKL..................
Os03g0197800_OsIDD1       YRCDCGTLFS..RRDSFITHRAFCDALAEESAKARAAPPPP...................
Os01g0195000_OsIDD2       YRCDCGTLFS..RRDSFITHRAFCDALAEESARAVT........................
Os09g0555700_OsIDD3       YRCDCGTLFS..RRDSFITHRAFCDALAQENAR.MPPIGAG...................
Os02g0672100_OsIDD4       YRCDCGTLFS..RRDSFITHRAFCDALAQESAR.LPPAAAG...................
Os07g0581366_OsIDD5       YRCDCGTLFT..RRDSFVTHRAFCGALVEETGRVLAVPAPPSPRPPDLEAEENVDKDKDK
Os08g0554400_OsIDD6       YRCDCGTLFS..RRDSFITHRAFCDALAQESGRIMPPMGAA...................
Os02g0518500_OsIDD7       YRCDCGTIFS..RKDSFITHRAFCDALAEDTSRVNHSLATMVG.................
Os01g0242200_OsIDD8       YRCDCGTLFS..RRDSFITHRAFCDALAEETARLNAASGAA...................
Os01g0935000_OsIDD9       YKCDCGTVFS..RRDSFVTHRAFCDALAQENNKLAQPMNMA...................
Os04g0566400_OsIDD10      YRCDCGTLFS..RRDSFITHRAFCDALAQESSR.LPPTSLSSLT................
Os01g0572300_OsIDD11      YKCDCGTIFS..RRDSFITHRAFCDALTEESAKAIGGI......................
Os08g0467100_OsIDD12      HTCDCGRVFS..RVESFIEHQDACNAGRPRAAEASSSPGRGGGVV...............
Os09g0449400_OsIDD13      HSCDCGRVFS..RVESFIEHQDTCNASRGQAAAVAEGGNVSTAAC...............
Os03g0237250_OsIDD14      HSCDCGRVFS..RVESFIEHQDACNSGRVRG.............................
Os01g0888700_OsIDDa       ELQNP.....DDVFDTILGLVVRLAEHGLIHCDFNEFNIMIDDDE...............
Os01g0871200_OsIDDb       WLCSCGTTFS..RKDKLFGHVALFQGHTPALPMDDIKVTGA...................
Os03g0838800_OsIDDc       WVCSCGTSFS..RKDKLFAHVAIFDGHSPALPPEDYDDDAA...................
Os04g0165200_ART2         WLCSCGTFFS..RKDKLMGHVALFAAGHAPVPVAP.........................
Os07g0162300_OsIDDe       DAALSDDKLRESYFELITTMRTLYQKCKLVHGDLSEYNILYFEG................
Os10g0419200_Ehd2         YRCDCGILFS..RKDSLLTHRAFCDALAEESARLLAAANNSS..................
Os08g0562300_OsIDDf       WLCSCGTSFS..RKDKLIGHVSLFAGHQPVMPLDAPRAGKR...................
Os02g0572900_STOP1        WRCSCGTTFS..RKDKLFGHLALFEGHTPAIAEPN...KGV...................
Os12g0170400_DART1        WLCSCGTSFS..RKDKLFAHVALFQGHAPALPPPPPPPTSG...................
```

Figure S1. Multiple alignment of *IDD*-like transcription factors in Arabidopsis, Tomato and Rice. highly conserved amino acids are indicated in red characters and boxes.

```
                                                180       190
                                                  .         .
Solyc01g005060_SlIDD12     .....................PRLQPITAACSS..........SDTNLP...........
Solyc01g007120             .....................KVTIIDFPQMVSVSHPNAQMYFDRDVECIY.........
Solyc01g099340_SlIDD10     .....................TTQPLATG..PELIST.TQMLNLPQIR............
Solyc02g062940_SlIDD17     ..............AEEDLKVQVDVSSTPQTPRTPPPSSEP.PAVPSPAAP.........
Solyc02g085580_SlIDD1      ..............NEEPKTQAVASSSPPPSPPAPPPATESQPPPPPPAAP.........
Solyc03g098070             ...............LRSESQSLHP....PCLSRTASSTPSPSSDTNLSTTPWS......
Solyc03g121660_SlIDD11     .....................ANPILPSQAGS........STSQMNFQ............
Solyc04g008500_SlIDD4      ...............LMN...TLGMNNNQYLYGSSSNINLGTFSKLGNNNNIT.......
Solyc04g056320_SlIDDb      ......................PAIENEKDVGVDINGGSGNGLMDIRTGSDNGFLD....
Solyc04g080130_SlIDD18     ..............NAEG.DLKVDEAINKTVSSS.........SSPPP............
Solyc05g054030_SlIDD5      ..................KECHDMQIPKPIFLPTTTTHIPPHDQYSKILP..........
Solyc06g062670_SlIDD2      .....................RNPTIFSPQMN........LQFQQPHY............
Solyc06g065440             ......................TSN.QGQP..MMQLQRLEI...................
Solyc06g072360_SlIDD9      ...............LRSESQSLIQLPSAPCLSRTASS.PSPSSDHTNNLITTT......
Solyc06g075250_SlIDD7      .....................STANLNFRN.EILLNGGFGNTNLQQSTGNPQF.......
Solyc07g053570_SlIDD8      .....................NPLAMANN....INYHFIGAPLGPNMAQHFSS.......
Solyc08g063040_SlIDD16     ...........................................PPNSS............
Solyc08g080850             .....................HLYVIDVSQSVDLDHPHALDFLREDCVHVS.........
Solyc09g007550_SlIDD13     .....................TTNNLNFN....QQQQQ..PVGVSQIG............
Solyc09g065670_SlIDD15     .................................LMHRAAATSTIANSA............
Solyc09g074780_SlIDD3      .....................MEHNMHSGQMHEIMSS.NASLGLSEFN............
Solyc10g084180_SlIDD14     .....................ATNLINFPNTATLLNQQ..QANLHGGF............
Solyc11g017140             ......................TSD.RGQTGEVTMKARQENYKVN.ASLGNEFQNP....
Solyc11g066420             ......................KQSAR.................................
Solyc11g069240_SlIDD6      .....................TAVNLNNLR.NQLINGGIS.TNLQQQQ...QM.......
AT1G03840_MGP/AtIDD3       .....................SFAATAGS...NLNYHYLMGTLIPSPSLPQPP.......
AT1G14580_BLJ/AtIDD6       ...............MAAGGGGGARHGFYG..GASS..ALS.HNHFGNNPNSG.......
AT1G25250_FAL/AtIDD16      ................TNHR..HLQQHTMGLDAPSRTTS.....TASFG...........
AT1G34370_AtSTOP1          ......................TSTQRGSSEGGNNNQGMVGFNLGSASNANQETTQ....
AT1G55110_AtIDD7           ..........................AMPNP.........IMIQASNS............
AT1G68130_AtIDD14          ................SNHRLHEQQQHTTNATQTASTAENNENGDLSIG...........
AT2G01940_SGR5/AtIDD15     ................EPPRPPQTAVTVPACSSRTASTVSTPSSETNYGGTVAVTT....
AT2G02070_RVN/AtIDD5       ...............LPSHHFPYGQNTNNSNNNASS.MILG.LSHMGAPQNLD.......
AT2G02080_IME/AtIDD4       ...............MTAASSGVGSGGIYGRLGGGS..ALS.HHHLSDHPNFG.......
AT2G24990_AtIDDa           .....................HLYIIDVSQSVDLDHPLALNFLREDCDHVS.........
AT3G13810_AtIDD11          .....................QNQNNNQPNP.........LLIHQSAS............
AT3G45260.1_BIB/AtIDD9     .....................APAYLNNALDVEVNHGNINQNHQQRQL............
AT3G50700_AtIDD2           ..............NPE.....ILTRKNP...............VPN.............
AT4G02670_AtIDD12          ........................................SSNLTNPN............
AT5G03150_JKD/AtIDD10      .................I...STTNLNFGNESNVMNNPNLPHGFVHRG............
AT5G22890_STOP2            ......................GSSKPPTITLK...........................
AT5G37350_AtIDDb           .....................HLYIIDVSQSVDLDHPLALNFLREDCDHVS.........
AT5G44160_NUC/AtIDD8       .....................GLAAAGAPGSVNLNYQYLMGTFIP....PLQP.......
AT5G60470_EGRET/AtIDD13    .....................PLAANSTIATVTDTNNPILIQSQLDQS............
AT5G66730_ENY/AtIDD1       ..............YPE.....TVTRKNPEIEQKSPAAVESSPSLPPSSPPSV.......
Os03g0197800_OsIDD1        ..............ADEDGTSASAGAPPPQAPLPPPAPVPAPAPPPPP............
Os01g0195000_OsIDD2        ......................AAAAVAGQQQHGG....GMLFSQVAD............
Os09g0555700_OsIDD3        ................VYGGAGN..MTLG.LTGMAAPQLPAGFPDQAGQ...........
Os02g0672100_OsIDD4        ...............HLYGSAGAANMALS..LSQVGSHLASTLQDHGHHHHHHG......
Os07g0581366_OsIDD5        EEEVKEKEKEKELEENEDSPVAEVDEPQPSQAVAEVPQQCAPSPPPPPPILQEHPQPVVA
Os08g0554400_OsIDD6        ................LYAAAGAG.MAIGGLTGMAASHQLQPFQDHSSA...........
Os02g0518500_OsIDD7        .................SLHGQQQDMFSHGVPSFSSSPTDMIANLASN............
Os01g0242200_OsIDD8        .....................AAAAAATATSLCGQSYLFAGSGGPGMAGMRPN.......
Os01g0935000_OsIDD9        .....................AVTSALQGQ...QQAHHPVADDDDAAGVKSPH.......
Os04g0566400_OsIDD10       ..............SHLYGASNAGNMALS..LSQVGSHLTTSLQDGGGHHHHP.......
Os01g0572300_OsIDD11       .....................PAAMAAPGHHHHHHHHHHQLLFSPPPV............
Os08g0467100_OsIDD12       ..........VGAPAAASSSQQQQLFAVPASLSRTASS.ASPSSDLVVSPVAWPAT....
Os09g0449400_OsIDD13       ..........GGVAVLEQEKQLDLQAPAAASLSRTASS.TSPSSDVVASPVAWP......
Os03g0237250_OsIDD14       ...............EVVPVATTLPVIRPAALRHHHHHPPPPPPELQLLP..........
Os01g0888700_OsIDDa        .....................KVTMIDFPQMVSVKHRNAQMFFDRDIECIY.........
Os01g0871200_OsIDDb        ......................SEQPQGSE.AMNTMVGSAGYNFPGSSSDDIPNLD....
Os03g0838800_OsIDDc        ......................SGQLPHAAGEAVGRTVDTNRFFS.....DGLMIK....
Os04g0165200_ART2          ......................EASPSTTTTGTTTDRSRTGLID................
Os07g0162300_OsIDDe        .....................HLYIIDVSQSVDLDHPSALEFLKEDCLHVT.........
Os10g0419200_Ehd2          .....................SITTTTCNNSNISSNNNNNNINSISNS............
Os08g0562300_OsIDDf        ......................QRSSSASVAGNIDDTTGIGMGAA...............
Os02g0572900_STOP1         ......................ATAAAEASISMMEEGG.VEANCDREEDEEGGFDP....
Os12g0170400_DART1         ......................RRRHKQEEPEFTWGGGGGNEFLDVKGIAGVGSGS....
```

Figure S1. Multiple alignment of *IDD*-like transcription factors in Arabidopsis, Tomato and Rice. highly conserved amino acids are indicated in red characters and boxes.

```
                                                                200                   210
                                                                  .                     .
Solyc01g005060_SlIDD12       .................................KLAIMPLPKNS............TNLE
Solyc01g007120               ..............................KFFAKRYNMSFDENENDSDGLEVDINEVGR
Solyc01g099340_SlIDD10       ................................NSN............MKIP..SIPLNMA
Solyc02g062940_SlIDD17       ..........EPLPPPGSP.STAVVSS.....DSPIQHP...ESPHTNSNGTITKHDIEE
Solyc02g085580_SlIDD1        ..........KSLMTPTVPPSTAVMSF.....ASSVQNRELRENPVTNSAGAATKQVVEE
Solyc03g098070               ..........STLLK..................KLSSAAAAANP...NMVKSCTNKHNLE
Solyc03g121660_SlIDD11       ................................FQPFNHEIPVTFSLKKEQQTGFNLRPEI
Solyc04g008500_SlIDD4        ...............KTTTTTTTTTSG..VQFDHHHHDNNLIGSS...TT.TTTTTNNPF
Solyc04g056320_SlIDDb        ...................RLMLDGFESIDSYCFQDLFESSSPNGLNTVPNTTGWFV...
Solyc04g080130_SlIDD18       ..........QPLTPSTSVLSPVLSIQ.....SSELPVEYQNQGGVQIQQAQGTTTPVST
Solyc05g054030_SlIDD5        ..................................NLDLELFTSPN..........YFNQN
Solyc06g062670_SlIDD2        ................................FNSHEQIQAATFPMKKEQQSSDFRHIEI
Solyc06g065440               ............................................................
Solyc06g072360_SlIDD9        ..........NTIP....................WPNFIINTPN...AMIK....EHNLE
Solyc06g075250_SlIDD7        .......................GSNLFRPEFMGLG.LDPAISHHHQPQLNNVDGQKPRI
Solyc07g053570_SlIDD8        ...............LFKPVSNNNN.......NNNNNENSDPMRR..........NQLSL
Solyc08g063040_SlIDD16       ................................TSHHLNLHTLQNF......QMKQEQQQQ
Solyc08g080850               ..............................DFFKKHGVAVMTIRELFDFIVDPTINDDSV
Solyc09g007550_SlIDD13       ................................TSFAQDFTSMTTVN..PLHQHQQ.KPRL
Solyc09g065670_SlIDD15       ................................AIADEPNFHLDQSSVFPFPANQRHFANP
Solyc09g074780_SlIDD3        ................................NFDPKNSLKALPQELLPIPFKSMTNNMA
Solyc10g084180_SlIDD14       ................................AGCRPDFGSYTTGNNNSLHQQQQQKPRL
Solyc11g017140               ...................GVVKECPYNPSSYFSPLNFDTSNLNGFQEFPRPPFDESDSS
Solyc11g066420               ............................................................
Solyc11g069240_SlIDD6        .......................SGNIS..QLMGLGSLDPSN......QLNLDHGQKSRL
AT1G03840_MGP/AtIDD3         ...............SFPFGPPQPQ.......HHHHHQFPITTNNFDHQDVMKPASTLSL
AT1G14580_BLJ/AtIDD6         ...............FTPLAAAGYNLN....RSSSDKFEDFVPQA...TNPNPGPT.NFL
AT1G25250_FAL/AtIDD16        ................................PLLHGLPLLR.PP........RPSNQHS
AT1G34370_AtSTOP1            ...................PGMTDGRICFEESFSPMNFDTCNFGGFHEFPRLMFDDSESS
AT1G55110_AtIDD7             ................................PHHHHHQTQQNIGFSSSS.....QNIIS
AT1G68130_AtIDD14            ................................PILPGHPLQRRQS........PPSEQQP
AT2G01940_SGR5/AtIDD15       ......PQPLEGRPIHQRISSSILTNSSNNLNLELQLLPLSSNQN........PNQENQQ
AT2G02070_RVN/AtIDD5         ...............HQPGDVLRLGSGGGGGGAASRSSSDLIAAN...ASGYFMQEQNPS
AT2G02080_IME/AtIDD4         ...............FNPL..VGYNLN....IASSDNRRDFIPQS...SNPN......FL
AT2G24990_AtIDDa             ..............................DFFKKHGVAVMTIRELFDFIVDPAITDENV
AT3G13810_AtIDD11            ................................HPHHHHQTQPTINVSSSSSSSHNHNIIN
AT3G45260.1_BIB/AtIDD9       ...............................NTTSSQLDQPGFNTNRNNIAFLG...QTL
AT3G50700_AtIDD2             ...........PVPAP.VDTESAKIKS.....SSTLTIK...QS....ESPKTPPEIVQE
AT4G02670_AtIDD12            ................................PNFQGHHFMFNKSSSLLFTSSP.LFIEP
AT5G03150_JKD/AtIDD10        ................................VHHPDINAAISQFGLGFGHDLSAMHAQG
AT5G22890_STOP2              ............................................................
AT5G37350_AtIDDb             ..............................DFFKKHGVAVMTIRELFDFIVDPTISDENV
AT5G44160_NUC/AtIDD8         ...............FVPQPQTNPN.......HHHQHFQPPTS............SSLSL
AT5G60470_EGRET/AtIDD13      ...............................STGTADLNVNNNHTTLFGQKFTNSNPTQQ
AT5G66730_ENY/AtIDD1         ........AIAPAPAISVETESVKIIS.....SSVLPIQ...NSPESQENNNHPEVIIEE
Os03g0197800_OsIDD1          ..........AAAPAPAAQPEQRD.RD.....AALDQFATPAPAP......APPPVTAPP
Os01g0195000_OsIDD2          ................................VLDHQAAMAMGGHGLMQELCLKREQQQQ
Os09g0555700_OsIDD3          .................PSASAGDVLRLGGGSNGASQFDHLMASS...SGSSMFRSQG..
Os02g0672100_OsIDD4          ...........ASPDLLRFGGSGGG.......AMAARLEHLLSSS....SASAFRPLPPP
Os07g0581366_OsIDD5          VVPNVDEQEVVAKPAVIAKIEVEDERDEEVCFQEVDRYKDAELEDSNLLDNDTPMLPCFL
Os08g0554400_OsIDD6          .................ITTAA..........NAAAQFDHLMATSSAAAGSPAFRAAQPT
Os02g0518500_OsIDD7          ................................DHNSDSHLRS....LSPYALVTRNTTLF
Os01g0242200_OsIDD8          ...............MMMPPQFDAA.......QLSWLYGN....................
Os01g0935000_OsIDD9          ...............LKMFPDVDNI.......VAAATAGNPLLPP...............
Os04g0566400_OsIDD10         ............SPELLRLGGAGGGGGAGGGSSIAARLDHLLSPS...GASAFRP.PQP
Os01g0572300_OsIDD11         ................................MAHHQELAALQEQ...QQQQHHQDVMQS
Os08g0467100_OsIDD12         ......GGGGPAMVSPRAAAPAPAGGSIAAFHHRFTDPALSPPTPCGGRRGGCHTTHSLE
Os09g0449400_OsIDD13         ......GAGAPSMPSPKAAA..........FRGRF.DMAPSPPPPSYDHYRGGAGAHNLE
Os03g0237250_OsIDD14         .................................ASTTAPLAAAFS...........SNST
Os01g0888700_OsIDDa          ..............................KFFRKRFHLSSEKCE.EQDGSDIDDDENSR
Os01g0871200_OsIDDb          ...................MKMADD....PRYFSPLSFDPC.FGGLDDFTRPGFDISENP
Os03g0838800_OsIDDc          ...................GSMEDE....RGSLSPMGLDYCEFDGIDLFAAAAFDF....
Os04g0165200_ART2            ............................................................
Os07g0162300_OsIDDe          ..............................DFFKKRGVAVMSVTELFNFVIDQNIADEDV
Os10g0419200_Ehd2            ................................NNLLITSSSSSPPLFLPFSTTPAENPNP
Os08g0562300_OsIDDf          ............................................................
Os02g0572900_STOP1           ...................KFFQEWMEELRG.......GAVAPN..WSAPSEAGH.....
Os12g0170400_DART1           ...................GGGDEFFS..AGSFGAMDFGFGQLDASLAMLLPSEQFAG..
```

Figure S1. Multiple alignment of *IDD*-like transcription factors in Arabidopsis, Tomato and Rice. highly conserved amino acids are indicated in red characters and boxes.

```
Solyc01g005060_SlIDD12     LGLKSS......................................................
Solyc01g007120             PQFSEINKNSGFLD..............................................
Solyc01g099340_SlIDD10     .GSMFSSSSG..................................................
Solyc02g062940_SlIDD17     TTAKSRLSGICSSSSCTGCTNSNCFTSLTGICSSSWSSSSNGSTSNSSIFSSLTGICSST
Solyc02g085580_SlIDD1      AAVVASLTGNCSSS..............................................
Solyc03g098070             LQLLTT......................................................
Solyc03g121660_SlIDD11     PPWLLVGGGGGPGP..............................................
Solyc04g008500_SlIDD4      FLQDTNQDHYN.................................................
Solyc04g056320_SlIDDb      ............................................................
Solyc04g080130_SlIDD18     TPVITISTAAASGP..............................................
Solyc05g054030_SlIDD5      THNFSS......................................................
Solyc06g062670_SlIDD2      PPWLITTNSQPFQL..............................................
Solyc06g065440             ............................................................
Solyc06g072360_SlIDD9      LQLLTT......................................................
Solyc06g075250_SlIDD7      PLWLDN.NMNPNPGND............................................
Solyc07g053570_SlIDD8      WNNIGVAQEQDQIGININNLR.......................................
Solyc08g063040_SlIDD16     NNDLS.SQLFIQNP..............................................
Solyc08g080850             DSYLEKVQERILAR..............................................
Solyc09g007550_SlIDD13     SLWLNQANNNMS................................................
Solyc09g065670_SlIDD15     PPPQLPISAAATGV..............................................
Solyc09g074780_SlIDD3      PGGMFSITSGNLFGNSPRSV........................................
Solyc10g084180_SlIDD14     SLWLDQQAANHNNP..............................................
Solyc11g017140             FSFLLSGSCEYPPHKAAKFMSSSEME..................................
Solyc11g066420             ............................................................
Solyc11g069240_SlIDD6      PLWLHHENSNYGNPND............................................
AT1G03840_MGP/AtIDD3       WSGGNINHHQQVT...............................................
AT1G14580_BLJ/AtIDD6       MQCSPNQGLLAQ................................................
AT1G25250_FAL/AtIDD16      PAFAYPF.....................................................
AT1G34370_AtSTOP1          FQMLIANACGFSPRNVGESVSDTSL...................................
AT1G55110_AtIDD7           ............................................................
AT1G68130_AtIDD14          STLLYPF.....................................................
AT2G01940_SGR5/AtIDD15     QKVKEP......................................................
AT2G02070_RVN/AtIDD5       FHDQQDHHHHHQQGF.............................................
AT2G02080_IME/AtIDD4       IQSASSQGMLNTT...............................................
AT2G24990_AtIDDa           DSYLEEVQRKVIER..............................................
AT3G13810_AtIDD11          SLHFDTNNGNTNNS..............................................
AT3G45260.1_BIB/AtIDD9     PTNVFASSSSPSPR..............................................
AT3G50700_AtIDD2           APKPTSLN.VVTS...............................................
AT4G02670_AtIDD12          ...SLSTAALSTPP..............................................
AT5G03150_JKD/AtIDD10      LSEMVQMASTGNHH..............................................
AT5G22890_STOP2            ............................................................
AT5G37350_AtIDDb           DSYLEEVQRKVIER..............................................
AT5G44160_NUC/AtIDD8       WMG.........................................................
AT5G60470_EGRET/AtIDD13    QPNALALSSPPSPR..............................................
AT5G66730_ENY/AtIDD1       ASRTIGFN.VSSSD..............................................
Os03g0197800_OsIDD1        PPPVAAPNDCVSSS..............................................
Os01g0195000_OsIDD2        QQQFAPSWLTAQQQ..............................................
Os09g0555700_OsIDD3        .SSSSSFYLANGAAHHAPAQD.......................................
Os02g0672100_OsIDD4        QQQPPAPFLLGAAP..............................................
Os07g0581366_OsIDD5        PSPSEAIGTDGSST..............................................
Os08g0554400_OsIDD6        SSSSSPFYLGG.................................................
Os02g0518500_OsIDD7        SNQISPKESG..................................................
Os01g0242200_OsIDD8        ............................................................
Os01g0935000_OsIDD9        ............................................................
Os04g0566400_OsIDD10       AFFLNAAAAAAATG..............................................
Os01g0572300_OsIDD11       PHQHQQQQQQRQQL..............................................
Os08g0467100_OsIDD12       LQLMPPRTTTTCAG..............................................
Os09g0449400_OsIDD13       LQLMPP.....FNA..............................................
Os03g0237250_OsIDD14       TTGSSS......................................................
Os01g0888700_OsIDDa        PSFLSIQKAAGSLD..............................................
Os01g0871200_OsIDDb        FSFLPSGSCSFGQQNGDS..........................................
Os03g0838800_OsIDDc        ............................................................
Os04g0165200_ART2          ............................................................
Os07g0162300_OsIDDe        DHYLEKIQQKMLEN..............................................
Os10g0419200_Ehd2          NQLLFLQQHQAAH...............................................
Os08g0562300_OsIDDf        ............................................................
Os02g0572900_STOP1         ............................................................
Os12g0170400_DART1         .........DHQEENGDK..........................................
```

Figure S1. Multiple alignment of *IDD*-like transcription factors in Arabidopsis, Tomato and Rice. highly conserved amino acids are indicated in red characters and boxes.

```
Solyc01g005060_SlIDD12     ............................................................
Solyc01g007120             ............................................................
Solyc01g099340_SlIDD10     ............................................................
Solyc02g062940_SlIDD17     RSSSSNGSTGSISVFPSLSGICSNSRSSSSNGSTQSSSIFSSSTGICSSSIFPSSTGICS
Solyc02g085580_SlIDD1      ...CSNG.....................................................
Solyc03g098070             ............................................................
Solyc03g121660_SlIDD11     ............................................................
Solyc04g008500_SlIDD4      ............................................................
Solyc04g056320_SlIDDb      ............................................................
Solyc04g080130_SlIDD18     ............................................................
Solyc05g054030_SlIDD5      ............................................................
Solyc06g062670_SlIDD2      ............................................................
Solyc06g065440             ............................................................
Solyc06g072360_SlIDD9      ............................................................
Solyc06g075250_SlIDD7      ............................................................
Solyc07g053570_SlIDD8      ............................................................
Solyc08g063040_SlIDD16     ............................................................
Solyc08g080850             ............................................................
Solyc09g007550_SlIDD13     ............................................................
Solyc09g065670_SlIDD15     ............................................................
Solyc09g074780_SlIDD3      ............................................................
Solyc10g084180_SlIDD14     ............................................................
Solyc11g017140             ............................................................
Solyc11g066420             ............................................................
Solyc11g069240_SlIDD6      ............................................................
AT1G03840_MGP/AtIDD3       ............................................................
AT1G14580_BLJ/AtIDD6       ............................................................
AT1G25250_FAL/AtIDD16      ............................................................
AT1G34370_AtSTOP1          ............................................................
AT1G55110_AtIDD7           ............................................................
AT1G68130_AtIDD14          ............................................................
AT2G01940_SGR5/AtIDD15     ............................................................
AT2G02070_RVN/AtIDD5       ............................................................
AT2G02080_IME/AtIDD4       ............................................................
AT2G24990_AtIDDa           ............................................................
AT3G13810_AtIDD11          ............................................................
AT3G45260.1_BIB/AtIDD9     ............................................................
AT3G50700_AtIDD2           ............................................................
AT4G02670_AtIDD12          ............................................................
AT5G03150_JKD/AtIDD10      ............................................................
AT5G22890_STOP2            ............................................................
AT5G37350_AtIDDb           ............................................................
AT5G44160_NUC/AtIDD8       ............................................................
AT5G60470_EGRET/AtIDD13    ............................................................
AT5G66730_ENY/AtIDD1       ...LSND.....................................................
Os03g0197800_OsIDD1        ....SSG.....................................................
Os01g0195000_OsIDD2        ............................................................
Os09g0555700_OsIDD3        ............................................................
Os02g0672100_OsIDD4        ............................................................
Os07g0581366_OsIDD5        ....SCG.....................................................
Os08g0554400_OsIDD6        ............................................................
Os02g0518500_OsIDD7        ............................................................
Os01g0242200_OsIDD8        ............................................................
Os01g0935000_OsIDD9        ............................................................
Os04g0566400_OsIDD10       ............................................................
Os01g0572300_OsIDD11       ............................................................
Os08g0467100_OsIDD12       ............................................................
Os09g0449400_OsIDD13       ............................................................
Os03g0237250_OsIDD14       ............................................................
Os01g0888700_OsIDDa        ............................................................
Os01g0871200_OsIDDb        ............................................................
Os03g0838800_OsIDDc        ............................................................
Os04g0165200_ART2          ............................................................
Os07g0162300_OsIDDe        ............................................................
Os10g0419200_Ehd2          ............................................................
Os08g0562300_OsIDDf        ............................................................
Os02g0572900_STOP1         ............................................................
Os12g0170400_DART1         ............................................................
```

Figure S1. Multiple alignment of *IDD*-like transcription factors in Arabidopsis, Tomato and Rice. highly conserved amino acids are indicated in red characters and boxes.

```
                                                                              220
Solyc01g005060_SlIDD12   ....................................................SHHEN.TI
Solyc01g007120           ...............................................KELAASGFTRKDQ
Solyc01g099340_SlIDD10   .................................................FNQLGTNSSN.
Solyc02g062940_SlIDD17   GSSIFASSTGIYSNSSSLSSNRSTSGSSIFTSLTGICSSSSNSSSNSTFASLFASSTASG
Solyc02g085580_SlIDD1    ..........................................SSSSNVFGSLFASSTASG
Solyc03g098070           ......................................TTSSSSP...AFDVSVSSKPNE
Solyc03g121660_SlIDD11   .............................................G...PGPPQ.PQPID
Solyc04g008500_SlIDD4    ......................................ANNNNIKPLMVHGLMQLPNLDH
Solyc04g056320_SlIDDb    ............................................................
Solyc04g080130_SlIDD18   ...........................................SSSKVFASVFPSSSSAQ
Solyc05g054030_SlIDD5    ...................................................FVDQSDHHH
Solyc06g062670_SlIDD2    .............................................GAINPSPSV.PLSNF
Solyc06g065440           ............................................................
Solyc06g072360_SlIDD9    ......................................TSTTTTSPCSPFDVSISSKSRD
Solyc06g075250_SlIDD7    .......................................FLVASSSSTTSNLPHHELVQI
Solyc07g053570_SlIDD8    ..........................EIHQINPLLHHTSAPAP.APVYDANTLLASSRSN
Solyc08g063040_SlIDD16   .............................................NGNSSNMFGAP.PPH
Solyc08g080850           ...............................................GDMTA.EEEIADS
Solyc09g007550_SlIDD13   ...........................................SQSSNLFGLPDH....H
Solyc09g065670_SlIDD15   ................................................TTHISLNPWEPQ
Solyc09g074780_SlIDD3    .................................ASSSSGLQLSSNIPSNFNYLQDNGKNR
Solyc10g084180_SlIDD14   ........................................LQISSSSNLFDSSSTGGFQE
Solyc11g017140           ............................................................
Solyc11g066420           ............................................................
Solyc11g069240_SlIDD6    .......................................FLSTP.STTTSTLP.HELVQV
AT1G03840_MGP/AtIDD3     ..................................IEDRMAPQPHSPQEDYNWVFGNANNH
AT1G14580_BLJ/AtIDD6     .........................................NNQSLMNHHGLISLG..DN
AT1G25250_FAL/AtIDD16    ....................................................NAS.SAPF
AT1G34370_AtSTOP1        ............................................................
AT1G55110_AtIDD7         ................................................NSNLHG.PMKQE
AT1G68130_AtIDD14        ....................................................VTNGSIEL
AT2G01940_SGR5/AtIDD15   ....................................................SHHHNHNH
AT2G02070_RVN/AtIDD5     .....................................LAGNNNIKQSPMSFQQNLMQFSH
AT2G02080_IME/AtIDD4     ......................................PNNNNQSFMNQHGLIQFDPVDN
AT2G24990_AtIDDa         ...............................................GEISV.EDEIADS
AT3G13810_AtIDD11        .............................................NNSNNHLHTFPMKKE
AT3G45260.1_BIB/AtIDD9   ........................................SASDSLQNLWHLQGQ.SSHQ
AT3G50700_AtIDD2         .............................................NGVFAGLFESSSASP
AT4G02670_AtIDD12        ................................................TAALSAT.....
AT5G03150_JKD/AtIDD10    ........................................LFPSSSSSLPDFSGHHQFQI
AT5G22890_STOP2          ............................................................
AT5G37350_AtIDDb         ...............................................GEISV.EDEIADS
AT5G44160_NUC/AtIDD8     ...................................QDIAPPQ...PQPDYDWVFGNAKAA
AT5G60470_EGRET/AtIDD13  ........................................STSDSVHNLWKLQEEECAHQ
AT5G66730_ENY/AtIDD1     .........................................HSNNNGGYAGLFVSSTASP
Os03g0197800_OsIDD1      ........................................VAPTSQSLLSSMFAPPSVAQ
Os01g0195000_OsIDD2      .............................................QQQLEAMAGAGNPAA
Os09g0555700_OsIDD3      ..........................FGPEDGQSQAGQGSLLHGKPAAFHDLMQLPVQHQ
Os02g0672100_OsIDD4      ..........................QGFGDGGDGSGPHGFLQGKP..FHGLMQLPDLQG
Os07g0581366_OsIDD5      ........................................TGSSVTNAIAPATTTSTFAG
Os08g0554400_OsIDD6      ...........................GGDDGQAHT...SLLHGKP.AFHGLMQLPEQQG
Os02g0518500_OsIDD7      .................................................FPLDGSVTSYP
Os01g0242200_OsIDD8      ..................................................GKLSSSNASE
Os01g0935000_OsIDD9      .............................................PLSMAGCMLSSLAAP
Os04g0566400_OsIDD10     ..........................QDFGDD.AGNGQHSFLQAKP..FHGLMQLPDLQG
Os01g0572300_OsIDD11     .............................................VQQNCGYAVKPEMAP
Os08g0467100_OsIDD12     ......................................GSLGGTPVATAVGYYASSSPHS
Os09g0449400_OsIDD13     ......................................GGAAAAPGGMGACFYAAAHQHH
Os03g0237250_OsIDD14     ....................................................HEQHATTM
Os01g0888700_OsIDDa      ...............................................KELAASGFTRKEQ
Os01g0871200_OsIDDb      ............................................................
Os03g0838800_OsIDDc      ............................................................
Os04g0165200_ART2        ............................................................
Os07g0162300_OsIDDe      ...............................................GDMVANDDEITPT
Os10g0419200_Ehd2        .................................................HQLLLPQFQQP
Os08g0562300_OsIDDf      ............................................................
Os02g0572900_STOP1       ............................................................
Os12g0170400_DART1       ............................................................
```

Figure S1. Multiple alignment of *IDD*-like transcription factors in Arabidopsis, Tomato and Rice. highly conserved amino acids are indicated in red characters and boxes.

	230
**Solyc01g005060_SlIDD12**	EE..........................AHLKLSIGS..................
**Solyc01g007120**	DELEK...
**Solyc01g099340_SlIDD10**	.......................MS....SATALLQQAAQMGA..............
**Solyc02g062940_SlIDD17**	SLPSQAPQFTDW........FQSVAPSPPPDIEPPSSMEPISLCLAMN............
**Solyc02g085580_SlIDD1**	SLPSQAPVFSDI........FRAMAPEHTLEMAPPSSTEPISLGLAMS............
**Solyc03g098070**	DH......................SAHNLQLSIGS..................
**Solyc03g121660_SlIDD11**	LSSS................IFQSSRFSDQDYTQS.HQHHQ..............
**Solyc04g008500_SlIDD4**	NNNNSS..................SSTTMFNLNFFQNN.................LS
**Solyc04g056320_SlIDDb**	....
**Solyc04g080130_SlIDD18**	HVNSSYS...............DILRAIETEQVMAVEPISLSLSSSLYLSN.......
**Solyc05g054030_SlIDD5**	HN........................NNYIVQNND...................
**Solyc06g062670_SlIDD2**	SSSS..................IFPATTRLDQQYSQSSHKDLN..............
**Solyc06g065440**	....
**Solyc06g072360_SlIDD9**	D.......................HNLQLSIGS..................
**Solyc06g075250_SlIDD7**	AAHN..............NTQQWFINGTGVGDDSGIGSSSSQLPS..............
**Solyc07g053570_SlIDD8**	APPVSYNN...............NNHINWDQFIGSKTSPINN..............
**Solyc08g063040_SlIDD16**	MSAT...................ALLQKAAQIGVTSSSHTANNMS..............
**Solyc08g080850**	VFIQS...
**Solyc09g007550_SlIDD13**	MVQIP..................SHNMFGTNQILNPTPTTTTT..............
**Solyc09g065670_SlIDD15**	IPLNP..................NHTHEENHHQITHPVPIKTE..............
**Solyc09g074780_SlIDD3**	VAQGQ.................GQLSSVPMSATALLQKAAQMGA..............
**Solyc10g084180_SlIDD14**	MIQMQ..................SQNVFGSSNSTSANFSIAEA..............
**Solyc11g017140**	....
**Solyc11g066420**	....
**Solyc11g069240_SlIDD6**	APHANMLGSSSLHKNNNNNNNNQWFVNDD..GGDAIITTSSSMLMP..............
**AT1G03840_MGP/AtIDD3**	GELIT.TS...................DSLITHDNNINIVQSKEN...............
**AT1G14580_BLJ/AtIDD6**	NNN.....................NHNFFNLAYFQDTK.................
**AT1G25250_FAL/AtIDD16**	ES..........................LELQLSIGM..................
**AT1G34370_AtSTOP1**	....
**AT1G55110_AtIDD7**	ES....................QHHYQNIPPWLISSNPNPNGN..............
**AT1G68130_AtIDD14**	QL..........................LPSRNCADE..................
**AT2G01940_SGR5/AtIDD15**	DT..........................TNLNLSIAP..................
**AT2G02070_RVN/AtIDD5**	DNHNSA..................PSN.VFNLSFLSGNNGVTSATSNPNAAAAAAVS
**AT2G02080_IME/AtIDD4**	INLKSSG..................TNNSFFNLGFFQENT................
**AT2G24990_AtIDDa**	VFMKS...
**AT3G13810_AtIDD11**	QQSND...............HIMNYHHSIIPPWLAPQPHALTSS...............
**AT3G45260.1_BIB/AtIDD9**	WLLNE.................NNNNNNNILQRGISKNQEEHEM..............
**AT3G50700_AtIDD2**	SIYTTS..................SSSKSLFASSSSIEPISLGLSTS............
**AT4G02670_AtIDD12**	....
**AT5G03150_JKD/AtIDD10**	PMTST..................NPSLTLSSSSTSQQTSASLQ..............
**AT5G22890_STOP2**	....
**AT5G37350_AtIDDb**	VFMKS...
**AT5G44160_NUC/AtIDD8**	SACID.NN..................N...THDEQITQN.ANAS..............
**AT5G60470_EGRET/AtIDD13**	WLLNE.................YMNNNKNIFHKGIFKNQEDEIK..............
**AT5G66730_ENY/AtIDD1**	SLYASS..................TASPSLFAPSSSMEPISLCLSTNPSL.........
Os03g0197800_OsIDD1	APQYADP...............IGVGAGGHQERAVPAKPPALCLAPN...........
Os01g0195000_OsIDD2	MYGS..................ARLDQEFIGSSTPESGGAQQAG..............
Os09g0555700_OsIDD3	QSGNGN.....................LLNLGFFS..GSNGG..............
Os02g0672100_OsIDD4	NGTGGPSP..................SGPGLYNLGYIANSANSSGTSSHGHASQGQMTN
Os07g0581366_OsIDD5	LFASVTT...............SSTPQSRSLRDLIGVDPTFLCLAIG............
Os08g0554400_OsIDD6	SNGGG.....................LLNLSYFS..GGNGGHHHHHQE..GRLVF
Os02g0518500_OsIDD7	YMS...................MNSPYMSATALLQKAAEMGA..............
Os01g0242200_OsIDD8	LTS...............................ATAA..............
Os01g0935000_OsIDD9	LSSPF..........................LPGCKLG..............
Os04g0566400_OsIDD10	NGAGGPGA..................PGPNLFNLGFFANNGNSSG.SSHEHASQGLMSN
Os01g0572300_OsIDD11	WPTT..................MPYDHHHPLLQPLCNANAAATA..............
Os08g0467100_OsIDD12	PAAALPSRQ...............PVADAMRLQLSIGFGGA..............
Os09g0449400_OsIDD13	PTGVSQCN....................DASTQLQLSIGR................
Os03g0237250_OsIDD14	TT..........................TKLQLSIGP..................
Os01g0888700_OsIDDa	VEMDK...
Os01g0871200_OsIDDb	....
Os03g0838800_OsIDDc	....
Os04g0165200_ART2	....
Os07g0162300_OsIDDe	VLVQTLD...
Os10g0419200_Ehd2	PSSPP.......................AYFDHLAFGGGGGVITG..............
Os08g0562300_OsIDDf	....
Os02g0572900_STOP1	....
Os12g0170400_DART1	....

	240
**Solyc01g005060_SlIDD12**	.............................SS...............PNHEVLNL.....
**Solyc01g007120**	...........................LIEGELENHSDSDDEGTADEEDEE........
**Solyc01g099340_SlIDD10**	...........................TVNNNMNSTLFNG...................
**Solyc02g062940_SlIDD17**	...........................HGSSVFGSTG......QERMQYAAAP......
**Solyc02g085580_SlIDD1**	...........................HSSSIFRPAG......QERRQYAPAP......
**Solyc03g098070**	.......................SDFNESSENANEKRSSDALRLKDEATEQLRV.....
**Solyc03g121660_SlIDD11**	...........................QGFMNPNPSLSGPT........SGA.......
**Solyc04g008500_SlIDD4**	TNSGILGDHHHHHHRHDNN........SNISCSIPSLYG...VQVLESSNTTFSSG...
**Solyc04g056320_SlIDDb**	....
**Solyc04g080130_SlIDD18**	...........................NRASLFQEHTGGHGHGHGQHYGSATM......
**Solyc05g054030_SlIDD5**	...........................IEVKEIIEEATTQVTRLKSEANEILKI.....
**Solyc06g062670_SlIDD2**	...........................LHHQNPNPNLRGPTLGYDSMGESGV.......
**Solyc06g065440**	....
**Solyc06g072360_SlIDD9**	.......................SDLSENNNNDTHRWRND........VEGLRH.....
**Solyc06g075250_SlIDD7**	...........................RVLLKEEEENKRNMSETISSMYYNNHHNE..T
**Solyc07g053570_SlIDD8**	....................AEQLSNTSSANISVPSLFSCQQQIALSSPSA........
**Solyc08g063040_SlIDD16**	...........................ATTFFSPTTST.....................
**Solyc08g080850**	...........................FIPKTLDHVKDAEADVQRIISGEDTGD.....
**Solyc09g007550_SlIDD13**	...........................TN...TNTNT........NTPATPN.......
**Solyc09g065670_SlIDD15**	...........................SIHFPISSSLPFYHDQHYPVFHRGSFS.....
**Solyc09g074780_SlIDD3**	...........................TASSSINSPMMQKSFVTSAMAGPDISITR..P
**Solyc10g084180_SlIDD14**	...........................YNSLYSDSQN........SQLTKQN.......
**Solyc11g017140**	....
**Solyc11g066420**	....
**Solyc11g069240_SlIDD6**	...........................RVLK.EEEENK.....TLSPMYYNN.......
**AT1G03840_MGP/AtIDD3**	.........................ANGATSLSVPSLFSSVDQITQDANAASVAVA...
**AT1G14580_BLJ/AtIDD6**	...........................NSDQTGVPSLFTN...................
**AT1G25250_FAL/AtIDD16**	.............................ARTSAQARHNEK.RETSLTKERAN......
**AT1G34370_AtSTOP1**	....
**AT1G55110_AtIDD7**	...........................NGNLFPPVASSVNT.....GRSSFP.......
**AT1G68130_AtIDD14**	.............................TSLSLSIGTMDQ.KTMSEVEKKSYE.....
**AT2G01940_SGR5/AtIDD15**	.............................SSSYQHYNNFDRIKEIMASEQIMKI.....
**AT2G02070_RVN/AtIDD5**	SGNLMISNHYDGENAVGGGEGSTGLFPNNLMSSADRISSGSVPSLFSSSMQSPNSA...
**AT2G02080_IME/AtIDD4**	...........................KNSETSLPSLYSTD..VLVHHREENLNAG...
**AT2G24990_AtIDDa**	...........................YIPKSLDAVNNPEADVAKITSGQDTGD.....
**AT3G13810_AtIDD11**	...........................N....PNPSNGGGG.....GGSLFS.......
**AT3G45260.1_BIB/AtIDD9**	...........................KNVISNGSLFSSEARNNTNNYNQNGG......
**AT3G50700_AtIDD2**	...........................HGSSFLGSN.........RFHA..........
**AT4G02670_AtIDD12**	....ALLQKATS.....
**AT5G03150_JKD/AtIDD10**	...........................HQTLKDSSFSPLF...SSSSENKQNK......
**AT5G22890_STOP2**	....
**AT5G37350_AtIDDb**	...........................YIPKSLDAVNNPEADVAKITSGQDTGD.....
**AT5G44160_NUC/AtIDD8**	.........................LTTTTTLSAPSLFSSDQPQNANANSN....V...
**AT5G60470_EGRET/AtIDD13**	...........................KGNIYSGSNPTDGNIASLFSYNQEAV......
**AT5G66730_ENY/AtIDD1**	...........................FGPTIRDPP.........HFLTPLPP......
**Os03g0197800_OsIDD1**	...........................ASSSLFTAPVP.....ADRQQFAPPPPPS...
**Os01g0195000_OsIDD2**	...........................LSFGFSSTSSAPPH........P.A.......
**Os09g0555700_OsIDD3**	VDQFNG.........GAGNGGQG....SIVTSSG..LAGNHGGGGGGFPSLYNSSE..P
**Os02g0672100_OsIDD4**	TDQFSE......GGGGGGGGGGS....ETSAAALFGAGGNFSGGDHHQVSPAGMYANDQ
**Os07g0581366_OsIDD5**	...........................APSSLFPQTNA.....SDPCSFAPPP......
**Os08g0554400_OsIDD6**	PDQFNGVAAGNGARAGSGEHGNS....GNNADSGSIFSGNMMGGGGGFSSLYSSSD..Q
**Os02g0518500_OsIDD7**	...........................KTSQDPISPLLLKSFPNNLTSSRDHMNIS..S
**Os01g0242200_OsIDD8**	...........................AKEAESVPSVFSNQQHAKPAAPTD........
**Os01g0935000_OsIDD9**	...........................VDAARDAAMVFPPPPPPAGSAAAI........
**Os04g0566400_OsIDD10**	.DQFSC......GAGGGGGLTRR....RRGSPAVTSLVATMFLLQGSTTIKRRCCR...
**Os01g0572300_OsIDD11**	...........................QSSATSAPPTTPQL........PAA.......
**Os08g0467100_OsIDD12**	......................RDDGNNNSSSSSGEVSASATRLKEAAHEQLRL.....
**Os09g0449400_OsIDD13**	........................DEVMGAAGTSDEASAAAT.AKEQAREQLRQ.....
**Os03g0237250_OsIDD14**	.......................AAVVAAASGGGGACAAAAGGEEEQQREEVRR.....
**Os01g0888700_OsIDDa**	...........................YIDQNAEEES.SDDDSTSEQDNEDGDDVA..V
**Os01g0871200_OsIDDb**	....
**Os03g0838800_OsIDDc**	....
**Os04g0165200_ART2**	....
**Os07g0162300_OsIDDe**	...........................YVKQCEADIVNMSLMQRPSFANEPTAD.....
**Os10g0419200_Ehd2**	...........................SSCNDDNSSIAGDVMVAAGGDSVSFG......
**Os08g0562300_OsIDDf**	....
**Os02g0572900_STOP1**	....
**Os12g0170400_DART1**	....

```
                                   250       260       270                           280
                                     .         .         .                             .
Solyc01g005060_SlIDD12     .....VMADKAYAEEARRNAKREMEYAEREFENAKR....................IRQQ
Solyc01g007120             .....DINNTNVKQLESLDLAKEDVN..................................
Solyc01g099340_SlIDD10     ......VQIPIQSNHDHDQNETQ......IGSILQG..................FGGSML
Solyc02g062940_SlIDD17     ...QPSMSATALLQKAAQMGATATN...SSLLRGLG....................ILSS
Solyc02g085580_SlIDD1      ...QPAMSATALLQKAAQMGAAATS...SSFLRGIG....................VMSS
Solyc03g098070             .....AISEKAYAEEARQQAKRQIELAEQEFANAKR....................IRQQ
Solyc03g121660_SlIDD11     .MASPHISATALLQKAAQMGATMSNKASTVSAVSSGPGPAM...........LMRPHQIH
Solyc04g008500_SlIDD4      ....PIMSATALLQKAAQMGSSSTS.......NISA...................TA...
Solyc04g056320_SlIDDb      ............................................................
Solyc04g080130_SlIDD18     ...QPALSATALLQKAAQMGSTTSN...NSFLRGLG....................LAMS
Solyc05g054030_SlIDD5      .....AMEEKAMAIEKRQEAKCLIELANLEMAKAME....................IRQS
Solyc06g062670_SlIDD2      .VSPVHISATALLQKAAQFGATISNKASAVTATAAYTG..T...........AKIPHNTH
Solyc06g065440             ............................................................
Solyc06g072360_SlIDD9      .....ALAEKVYAEETRKEARREIELAEEELSNARR....................IRRQ
Solyc06g075250_SlIDD7      TAPATHMSATALLQKAAQMGSTRSN..SALFGTGFG..................LMG.SS
Solyc07g053570_SlIDD8      .....NMSATALLQKAAQIGATTTT........DPS...................IFLGN
Solyc08g063040_SlIDD16     .TTGIAMSGSGSGAGVARQYHPFE..NNNNNNNT...................NTDFVTG
Solyc08g080850             .....MYY.KTITGLKQALTATNSSAEDQQHLQIDELGQETAAAAETNKPSD........
Solyc09g007550_SlIDD13     ..PPIPMSATALLQKAAQMGSTKST.P.NYFSNTFG..................VMHSSS
Solyc09g065670_SlIDD15     .IPSPQLAATALLQRATTMGTNSTVNHVNSTMAH....................LNRGTL
Solyc09g074780_SlIDD3      SPNYDNFQMQQDSNNHQSQSQNQN.....LAAVIND..................MGIYSG
Solyc10g084180_SlIDD14     ..STTPMSATALLQKAAQMGSTKSN.QSTFFSNNFS..................KLNSSS
Solyc11g017140             ............................................................
Solyc11g066420             ............................................................
Solyc11g069240_SlIDD6      ....SHMSATALLQIAAQIGSTRSNNNSPLFSNQFG..................LMTSSS
AT1G03840_MGP/AtIDD3       .....NMSATALLQKAAQMGATSSTSPTTTITTDQS...................AYLQS
AT1G14580_BLJ/AtIDD6       ....................GADNN.......GPSA...................LLRGL
AT1G25250_FAL/AtIDD16      .......EEARKAEETRQEAKRQIEMAEKDFEKAKR....................IREE
AT1G34370_AtSTOP1          ............................................................
AT1G55110_AtIDD7           .HPSPAMSATALLQKAAQMGSTKSTTPEEEERSS...................RSSYNNL
AT1G68130_AtIDD14          .....KGETSLEREEARRETKRQIEIAELEFAEAKR....................IRQH
AT2G01940_SGR5/AtIDD15     .....AMKEKAYAEEAKREAKRQREIAENEFANAKK....................IRQK
AT2G02070_RVN/AtIDD5       ....PHMSATALLQKAAQMGSTSSN.......NNNG...................SN...
AT2G02080_IME/AtIDD4       ....SNVSATALLQKATQMGSVTSN.......DPSA...................LFRGL
AT2G24990_AtIDDa           .....MLY.QTITGLKDALPKIE.....EQKIEVNDE.......................
AT3G13810_AtIDD11          .LASPAMSATALLQKAAQMGSTKTPPLPPTTAYE...................RSTHNNN
AT3G45260.1_BIB/AtIDD9     ..QIASMSATALLQKAAQMGSKRSS.SSSSNSKTFG..................LMTS.I
AT3G50700_AtIDD2           ...QPAMSATALLQKAAQMGAASSG...GSLLHGLG....................IVSS
AT4G02670_AtIDD12          .LSSTTFGGGGQTR...SIGHHRHLTNVNEFLG.....................VDRVMM
AT5G03150_JKD/AtIDD10      ..PLSPMSATALLQKAAQMGSTRSN.SSTAPSFFAG..................PTMTSS
AT5G22890_STOP2            ............................................................
AT5G37350_AtIDDb           .....MLY.QTITGLKDALPKVD.....EQQIEVNAE.......................
AT5G44160_NUC/AtIDD8       .....NMSATALLQKAAEIGATSTT...TAATNDPS...................TFLQS
AT5G60470_EGRET/AtIDD13    ..NMASFSATTLLQKVAQTGTP..S.SSETSTTMFG..................QMTSSI
AT5G66730_ENY/AtIDD1       ...QPAMSATALLQKAAQMGSTGSG...GSLLRGLG....................IVST
Os03g0197800_OsIDD1        ..PSPHMSATALLQKAAQMGATSSS...SSFLRCLG....................LDMS
Os01g0195000_OsIDD2        .ASSAHMSATALLQKAAQMGATLSRPSSHAHMAA...................AAAASTH
Os09g0555700_OsIDD3        AGTLPQMSATALLQKAAQMGATTSSYNAGGAGGASS...................LLRGA
Os02g0672100_OsIDD4        AMMLPQMSATALLQKAAQMGSSTSS....ANGAGAS...................VFGGG
Os07g0581366_OsIDD5        ...APHMSATALLQKAAEVGASQSS...SSFLKEFG....................LAAS
Os08g0554400_OsIDD6        TVPPPQMSATALLQKAAQMGATTSS...GGAGSVNS...................LLRGL
Os02g0518500_OsIDD7        RSQGDSLGNSTVNSDCMKTTEDENSYMIGRGNILIN..................APWSSG
Os01g0242200_OsIDD8        ......MSATALLQKAAQIGAVTST......AAMPL...................VSPFE
Os01g0935000_OsIDD9        ......MSATALLQKAAELGATTST......GCYGG...................VAFPA
Os04g0566400_OsIDD10       .....RCRRPALLQKAAQMG.ATSS....ANGPGS....................MFRG.
Os01g0572300_OsIDD11       .AAAAHLSATALLQKAAQMGATIGGAGTGAAGAH...................YAHMASP
Os08g0467100_OsIDD12       .....AMAEKAAADEARAQAKRQAELADQELATARR....................MRYQ
Os09g0449400_OsIDD13       .....AMAEKEAAGEARAQARRQVELAEQELATARR....................MRHQ
Os03g0237250_OsIDD14       .....ALEEKTAADAARERAREEAAAAERALEDARR....................ARHR
Os01g0888700_OsIDDa        KIGSLKIAEQDSAEVPDCTLASKDSNEPETFAKENETSTSCSGENNSINPSP........
Os01g0871200_OsIDDb        ............................................................
Os03g0838800_OsIDDc        ............................................................
Os04g0165200_ART2          ............................................................
Os07g0162300_OsIDDe        .....KLYNQPLLGFVRNKNEPTK....NQQVQSEEP.......................
Os10g0419200_Ehd2          .....LTSEGSVTMHAGDVGRRRLTRDFLGVDHDAG...................EVDEL
Os08g0562300_OsIDDf        ............................................................
Os02g0572900_STOP1         ............................................................
Os12g0170400_DART1         ............................................................
```

```
Solyc01g005060_SlIDD12      AQAEI.......................................................
Solyc01g007120              ............................................................
Solyc01g099340_SlIDD10      QNNGDD..............HHKSSRV.................................
Solyc02g062940_SlIDD17      SS..................SSSGQHEWNGRQID..........................
Solyc02g085580_SlIDD1       TS..................SSNGHQEWSGRPSD..........................
Solyc03g098070              AQAEL.......................................................
Solyc03g121660_SlIDD11      VSATATAAESVSNATDFGLNLSSREDLPTGFFNSLAS.......................
Solyc04g008500_SlIDD4       SLFKA..............FGSNTSGS.................................
Solyc04g056320_SlIDDb       ............................................................
Solyc04g080130_SlIDD18      PS..................TSTPEENSLARRTN..........................
Solyc05g054030_SlIDD5       VCASS.......................................................
Solyc06g062670_SlIDD2       VSVTST..DSATKQTQQ..KLSSREDLTT...............................
Solyc06g065440              ............................................................
Solyc06g072360_SlIDD9       AQMEL.......................................................
Solyc06g075250_SlIDD7       FSKSNGQ...........GQFATHDQN.................................
Solyc07g053570_SlIDD8       FSMNNCSNNNNNKNNDNNKFCGFYVST.................................
Solyc08g063040_SlIDD16      NTSFPDF............GASVTPG..................................
Solyc08g080850              ............................................................
Solyc09g007550_SlIDD13      SPSSNN.............TTQIFNNN.................................
Solyc09g065670_SlIDD15      THVTPNG............FLSFGSEN.................................
Solyc09g074780_SlIDD3       ILMSND..............QNNDGGY.................................
Solyc10g084180_SlIDD14      SSSPNTP...........TFTSLQSCE.................................
Solyc11g017140              ............................................................
Solyc11g066420              ............................................................
Solyc11g069240_SlIDD6       LSMKN................SSSSSS.................................
AT1G03840_MGP/AtIDD3        FASKS.NQIVEDGGSDR.FFASFGSNS.................................
AT1G14580_BLJ/AtIDD6        TSSS...............SSSVVVND.................................
AT1G25250_FAL/AtIDD16       AKTEL.......................................................
AT1G34370_AtSTOP1           ............................................................
AT1G55110_AtIDD7            ITTTMAA............MMTSPP...................................
AT1G68130_AtIDD14           ARAEL.......................................................
AT2G01940_SGR5/AtIDD15      AQAEL.......................................................
AT2G02070_RVN/AtIDD5        TNNNN..............NASSILRS.................................
AT2G02080_IME/AtIDD4        ASSSN..............SSSVIANH.................................
AT2G24990_AtIDDa            ............................................................
AT3G13810_AtIDD11           LTTTMAA............MMTSPSGFIS...............................
AT3G45260.1_BIB/AtIDD9      FNNKQ.......................................................
AT3G50700_AtIDD2            TS..................TSIDA...................................
AT4G02670_AtIDD12           TSASSSE............YDQLVVDG.................................
AT5G03150_JKD/AtIDD10       SATASP.................PPRS.................................
AT5G22890_STOP2             ............................................................
AT5G37350_AtIDDb            ............................................................
AT5G44160_NUC/AtIDD8        FPLKSTDQTTSYDSGEK.FFALFGSNN.................................
AT5G60470_EGRET/AtIDD13     FNNTM.......................................................
AT5G66730_ENY/AtIDD1        TS..................SSMELSNH................................
Os03g0197800_OsIDD1         SSSSAP.............PSSSGQQQQHHHHHH......QETMQVPLPASSLP......
Os01g0195000_OsIDD2         NSSSSAA............TTNAPPPP.................................
Os09g0555700_OsIDD3         SSHG...............ISVGEGP..................................
Os02g0672100_OsIDD4         FAGSSAPSSIPHGR.GTTMVDQGQMHL.................................
Os07g0581366_OsIDD5         TSSSPPSKLSQGRFTTGNTPTTSHPHPHPHPHPHPPQGRFMDNVPQPPPPAKLPHRMFTD
Os08g0554400_OsIDD6         GSGGGGGALNGKPAGAAGFIMSGESSS.................................
Os02g0518500_OsIDD7         IMRPGT..............VPLIG...................................
Os01g0242200_OsIDD8         PTKPGGATASPAD..ECGKFDGAALFA.................................
Os01g0935000_OsIDD9         MGIAGGLDRLPAIGHHLAPYDDVVVPA.................................
Os04g0566400_OsIDD10        FVGSS.....PHMRPAAQHMDQSDAHL.................................
Os01g0572300_OsIDD11        AGAGAPA............GGSATFGL.................................
Os08g0467100_OsIDD12        AQVEL.......................................................
Os09g0449400_OsIDD13        AQVEL.......................................................
Os03g0237250_OsIDD14        ARGEL.......................................................
Os01g0888700_OsIDDa         ............................................................
Os01g0871200_OsIDDb         ............................................................
Os03g0838800_OsIDDc         ............................................................
Os04g0165200_ART2           ............................................................
Os07g0162300_OsIDDe         ............................................................
Os10g0419200_Ehd2           ELDELP......................................................
Os08g0562300_OsIDDf         ............................................................
Os02g0572900_STOP1          ............................................................
Os12g0170400_DART1          ............................................................
```

```
                                        290       300       310        320
                                          .         .         .          .
Solyc01g005060_SlIDD12      ...............ERAKQLKEEAINRISSSILEITCHACKNK.FQTIDN.........
Solyc01g007120              ..............HQTGDDKHDDNHQVGDENDPEEEEEAESEDDPELEKSLS.......
Solyc01g099340_SlIDD10      ..............LQNEQGWFNNNNNNSNTGLFNEKQRTLNKEAG..HSNEE.......
Solyc02g062940_SlIDD17      ................TDGATLAAG......LGLGLPCD...DGSGLKELMLG.......
Solyc02g085580_SlIDD1       ................ANGASLAAG......LGLGLPCD...AGSGLKELMLG.......
Solyc03g098070              ...............DKANALKEHAIKQINSTLSQITCHSCKQK.FQSTTR.........
Solyc03g121660_SlIDD11      ........YGNKAANPSAVITPVTIPLSTAPHPTPSTLQDMMNSFSSVNPTGFEG.....
Solyc04g008500_SlIDD4       ..............SSSGTKSDHQAFNFGSDITDSGIINA..YGGGSHEGY.........
Solyc04g056320_SlIDDb       ............................................................
Solyc04g080130_SlIDD18      ................ALGHVKLESNINAEVPGLGIQLH...GHSGFTDLMMGPSIS...
Solyc05g054030_SlIDD5       ...............SSS...........SHVMKIIKCSSCNNKQFQSVSS.........
Solyc06g062670_SlIDD2       ........ITGPANNISGIMTSFSNGFDGSTMFEDAILFGGFNNLNSKKEN.........
Solyc06g065440              ............................................................
Solyc06g072360_SlIDD9       ...............EKAQVLKEEAIRKINFTLSQITCYACKQK.FQPKKT.........
Solyc06g075250_SlIDD7       ..............FNGLMMHSPNNNNS...NQGNRLLFGDMNSTSLEGNASGKNSDPFN
Solyc07g053570_SlIDD8       ..............PTTNNSISNSLGSDVDQSSSVNDFSA..TIHPIQMYPPPKRRH...
Solyc08g063040_SlIDD16      ..............FMEQVHDMQNMMATTAPSLPCTSFDEGFGRGAHLMRQ.........
Solyc08g080850              ..............GESETETESDDDDDDESDCSEGSSSDGEKPTAADKKAAR.......
Solyc09g007550_SlIDD13      ..............EIHQAFAKQTEDYN...QTENILINGCPNMNSSVNSKAINL....D
Solyc09g065670_SlIDD15      ..............IPGNWQKRDNLTRDFLGLTGDHHHHHDHDDSSSSAGGVGGG.....
Solyc09g074780_SlIDD3       ..............IKNVTEVLDRDNHNHST...NDRVRNQTMMVQ..NGSND.......
Solyc10g084180_SlIDD14      ..............TTDQIFAKKSEDYTTSLLQSNNGLFGCPNFTSSGINKSINL....D
Solyc11g017140              ............................................................
Solyc11g066420              ............................................................
Solyc11g069240_SlIDD6       ..............ASNVAIHENVQGN.......NGLLIGATNSTTFVANNAN......T
AT1G03840_MGP/AtIDD3        ...............VELMSNNNNGLHEIGNPRNGVTVVS..GMGELQNYPWKRRR....
AT1G14580_BLJ/AtIDD6        ..............FG......DCDHGNLQGLMNSLAATT..DQQGRSP...........
AT1G25250_FAL/AtIDD16       ...............EKAHVVREEAIKRINATMMEITCHSCKQL.FQLP...........
AT1G34370_AtSTOP1           ............................................................
AT1G55110_AtIDD7            ...............EPGFGFQDYYMMNHQHHGGGEAFN...GGFVP.............
AT1G68130_AtIDD14           ...............HKAHLFREEASRRISATMMQITCHNCKQH.FQAPAALVPPPP...
AT2G01940_SGR5/AtIDD15      ...............ERAKFLKEQSMKKISSTIMQVTCQTCKGQ.FQAVAVP........
AT2G02070_RVN/AtIDD5        ..............FGSGIYGENESN..LQDLMNS.FSNP..GATGNVNGV.........
AT2G02080_IME/AtIDD4        ..............FGGGRIMENDNNGNLQGLMNSLAAVN..GGGGSGG...........
AT2G24990_AtIDDa            ...............EKEEEGEEEEDGESEEGSEEEESE..EELGHEDKKAAR.......
AT3G13810_AtIDD11           ............SNNNNHVLFQDYNASGFDNHGREEAFDDTFGGFLRTNEV.........
AT3G45260.1_BIB/AtIDD9      ..............AEN.IKTKEVDERG................................
AT3G50700_AtIDD2            ....................IVPHG......LGLGLPCG.GESSSGLKELMMG.......
AT4G02670_AtIDD12           ..............FTSTWQKADRLTRDFLGLT...........................
AT5G03150_JKD/AtIDD10       ..............SSPMMIQQQLNNFNTNVLREN..HNRAPPPLSGVSTSSVDN....N
AT5G22890_STOP2             ............................................................
AT5G37350_AtIDDb            ...............EEE...EEEEEGSGEES..EEESE..KELGPEDKKAAR.......
AT5G44160_NUC/AtIDD8        ..............NIGLMSRSHD.HQEIENARNDVTVAS..ALDELQNYPWKRRR....
AT5G60470_EGRET/AtIDD13     ..............LNSYCLTAKNNEEE................................
AT5G66730_ENY/AtIDD1        .................DALSLAPG......LGLGLPCSSGGSGSGLKELMMG.......
Os03g0197800_OsIDD1         ....EWPPR....LQPEPSPMLSSG......LGLGLPYDATGGPVSLPELMMG.......
Os01g0195000_OsIDD2         ..............PTSNVSSTCVGAGGYGLAFEASHFIAADESSRGARSD.........
Os09g0555700_OsIDD3         .....................ANERSSYQNLIMGSMASGG..GGAGFAG...........
Os02g0672100_OsIDD4         ..............QSLMNSLAGGGNADHQGMFG........SGSMIDPRLYDMDQH...
Os07g0581366_OsIDD5         NSVQQWHHRSNQQMEMEPGPMLPGG......LGLGLTYDT..GNSGLPDLMMG.......
Os08g0554400_OsIDD6         ..............RSTASQTAENESQLRELMMNTLSATG..GGTGAGT...........
Os02g0518500_OsIDD7         ..............LMNHPFSMREEKDNPSIFPENQTQHNRQDNISGVVDADA.......
Os01g0242200_OsIDD8         ..............AASHHNAN......LGGAMS.ELTGA..AGNVPYDVLSAVRH....
Os01g0935000_OsIDD9         ..............ALQGQTATQLVGFDLGGLLPGQLYGG..GGGAMTRAIGSLMHG...
Os04g0566400_OsIDD10        ..............NDLMNSLAGGG.VNAAAMFGGTNGGGVPGAGMFDPRLCDI.EH...
Os01g0572300_OsIDD11        ..............GLSCLNTHQDGGGGGGNGLIPAGMMGHLARTASHGRS.........
Os08g0467100_OsIDD12        ...............SRAHVLRDHAIRQVDATQLQITCYSCSHK.FRARAA.........
Os09g0449400_OsIDD13        ...............SRAHALRDHAVRQVNATLLQITCFSCRHK.FRAAAAGAPLP....
Os03g0237250_OsIDD14        ...............EKALALRDHAAR....LIAQVTCHACRQR.SLAVMS.........
Os01g0888700_OsIDDa         ..............SSNGDAKEPTESQDNDDSDDDSSDDPDGEDDDALAKQLN.......
Os01g0871200_OsIDDb         ............................................................
Os03g0838800_OsIDDc         ............................................................
Os04g0165200_ART2           ............................................................
Os07g0162300_OsIDDe         ..............LDLQNKCSSEHSESCTSSDEDGSWHEILKVGPEERKAAR.......
Os10g0419200_Ehd2           ................ADLSTTAAACQGCNFAAATTAACCATDFTTGSRQYLG.......
Os08g0562300_OsIDDf         ............................................................
Os02g0572900_STOP1          ............................................................
Os12g0170400_DART1          ............................................................
```

330

**Solyc01g005060_SlIDD12**	..................NVDSSPPMSYMS...SDGE.................REQ...
**Solyc01g007120**	.................KQRKKAIQAAHRGKRNFASRNTYKDKGGKSSQNSKVHKQLSGW
**Solyc01g099340_SlIDD10**	......................SLTLDFLGIGG.....MRHR...NLHEMHQHQQEMSFE
**Solyc02g062940_SlIDD17**	..............TPSVFGPKHPTLDLLGLGMA.AGGGSTP..GLSALITSMGGNLDVA
**Solyc02g085580_SlIDD1**	..............TPSVFGPKHPTLDLLGLGMA.ASVGPSP..GLSALLTSMGSNLDMV
**Solyc03g098070**	..............VSIADENNSLALSYISSGLSEGHHHE........VAANNINQNEHL
**Solyc03g121660_SlIDD11**	.SNFEDPFSILNPKNNNGSGNDDMTKDFLGLRPLSHS.DIFNIAGLVNS........TP.
**Solyc04g008500_SlIDD4**	...NTTTKLNFEQNQGPPQKKQ.LTRDFLGV............GEIVRSMNG..RFSTQR
**Solyc04g056320_SlIDDb**	....
**Solyc04g080130_SlIDD18**	............HQTSMMFGSKPPTLDFLGLGNG.ASG..TPSNGFSAFLSSMEDGFGVV
**Solyc05g054030_SlIDD5**	.................SKDATLTNNYYLSSSIYRR........................
**Solyc06g062670_SlIDD2**	....EDEDQQLYFNGSMNEEDHILTKDFLGLKPLSHTDDIFNIAALVN............
**Solyc06g065440**	....
**Solyc06g072360_SlIDD9**	..............TTTTTR..............................TTSIDQNIHL
**Solyc06g075250_SlIDD7**	LMPRNNKGKQVNLSGNEA.MEGGLTRDFLGVGGN.......ESRPFLSK.NELAKFGNIS
**Solyc07g053570_SlIDD8**	...IQIEDSVKGVEVGAGGGAGGQTRDFLGVG..............VQSICHPSSINGLI
**Solyc08g063040_SlIDD16**	.........GKENN.......EGLTRDFLGLRAFPHHRDFLSTTTPPAALHYNMGSSAAA
**Solyc08g080850**	.................KENKKKVKEEKREARKHKVPKAVKKKKKKLAKAKKYR......
**Solyc09g007550_SlIDD13**	QALLQTGGMHN.QNVHFN.LHNSLTRDFLGMGNE.......GGQPFLPQ..ELAKFASIN
**Solyc09g065670_SlIDD15**	.......GGGGQVLPTNGNVGNGLLLSYGGNRMQDFQQTLYERDNSVLKHHPLGFGFAGA
**Solyc09g074780_SlIDD3**	......................MLTVDFLGIGG.....ASRAPISNLQEQRRFEAAINQQ
**Solyc10g084180_SlIDD14**	EAIMQTVGMHNTQAVSMNGMDNCLTRDFLGIKHE.......GNCQFLPQ..EIVKFASMN
**Solyc11g017140**	....
**Solyc11g066420**	....
**Solyc11g069240_SlIDD6**	LMMMQDKGK...........QGNLTRDFLGGGRN.......EKRLFLQQTHEMAKFENNK
**AT1G03840_MGP/AtIDD3**	............VDIG.NAGGGGQTRDFLGVG..............VQTICHSSSINGWI
**AT1G14580_BLJ/AtIDD6**	....SLFDLHFANNLSMGGSDR.LTLDFLGVN...........GGIVSTVN......GRG
**AT1G25250_FAL/AtIDD16**	..............VTADESTSSLVMSYVSSATTEGE.................CE....
**AT1G34370_AtSTOP1**	....
**AT1G55110_AtIDD7**	..........GEEKNDVVDDGGGETRDFLGLRSLMSHNEILSFANNLGN........CLN
**AT1G68130_AtIDD14**	.............QTHCTDESTSLAVSYMSSATTEGE.................KASDRA
**AT2G01940_SGR5/AtIDD15**	...............AATADETSLVVSYMSSANTDGE.................LENGF.
**AT2G02070_RVN/AtIDD5**	...DSPFGSYGGVNKGLSADKQSMTRDFLGV............GQIVKSMSGSGGFQQQQ
**AT2G02080_IME/AtIDD4**	....SIFDVQFGDNGNMSGSDK.LTLDFLGV............GGMVRNVNRGGGGGGRG
**AT2G24990_AtIDDa**	.................KEHKKKVKEEKRESRKTKTPKSVKKRKKKVSKPHKTR......
**AT3G13810_AtIDD11**	......TAAAGSEKSTKSGGGEGLTRDFLGLRPLMSHNEILSFAG.LGS........CIN
**AT3G45260.1_BIB/AtIDD9**	.......................FTRDFLGVGSQHRSWPLLMVNHNLPDSSPPASTDGTP
**AT3G50700_AtIDD2**	..............NSSVFGPKQTTLDFLGLGRA.VGNGNGPSNGLS...TLVGGGTGID
**AT4G02670_AtIDD12**	.......GHGGHVS.....VRPGDMLEYAGG..VAFPMSAYDTES.....HDHSFQKAYD
**AT5G03150_JKD/AtIDD10**	PFQSNRSGLNP..AQQMG.....LTRDFLGVSNEHHP.HQTGRRPFLPQ..ELARFAPLG
**AT5G22890_STOP2**	....
**AT5G37350_AtIDDb**	.................KEHKKKVKEEKRESRKTKTPKSVKKRKKKVSKPHKTR......
**AT5G44160_NUC/AtIDD8**	............VDGGGEVGGGGQTRDFLGVG..............VQTLCHPSSINGWI
**AT5G60470_EGRET/AtIDD13**	.......................LTRDFLGVGSSED..QQRLHRHRFPSSVPLTANHDIP
**AT5G66730_ENY/AtIDD1**	..............NSSVFGPKQTTLDFLGLGRA.VGNGGNTGGGLSALLTSIGGGGGID
**Os03g0197800_OsIDD1**	..............QSTLFSAKPATLDFLGLGVS.PTGASTSRG.FPTFIQPIGGAVSLA
**Os01g0195000_OsIDD2**	.........RDTGNGVAGAGNDGLTRDFLGLRAFSHG.NIMSMAGFDP....CMSTTSAS
**Os09g0555700_OsIDD3**	.....SFSGASGFGGAVDDGKLS.TRDFLGVGVV.........QGISGSAAMGPPRHG.A
**Os02g0672100_OsIDD4**	...EVKFSLQRGGGGG...GDGDVTRDFLGVGG..........GGFMRGMSMARGEHHGG
**Os07g0581366_OsIDD5**	..............PSALYGPKPATLDFLGLGIGGTMGGSTANGGLPALM..VGGELDMG
**Os08g0554400_OsIDD6**	.....VFVGG.GFPG.VDDGKLS.TRDFLGV...............SGGAPGLQLRHGGA
**Os02g0518500_OsIDD7**	......................GLTQDFLGLGGSGT..LDMSSETYNADVTALSYSDEQE
**Os01g0242200_OsIDD8**	..........HAGLKDAGGVGREETRDFLGVG..............VQALCS.SSIHGWI
**Os01g0935000_OsIDD9**	.......GDQHAGVVDRRRGEGVRVVDYMGVDDDDDH.....GCFDGVGPFG.PHIGPWA
**Os04g0566400_OsIDD10**	...EVKFSQGGGGGGGAGAGTGDGTRDFLGVGG..........GGIVHGMSTPRGDHHQS
**Os01g0572300_OsIDD11**	.........GEDVAGAGGGGGDGMTRDFLGLRAFSHR.DIL.LAGFDSS...CMGHVNAA
**Os08g0467100_OsIDD12**	..............AMISSDVAS.YVSSVVTEGGDAEVDNDGNLHRRRLNADDGMPRGHS
**Os09g0449400_OsIDD13**	..............AAMSSDVACSYVSSVVTEGGDADDP.........LDVVDATRRRLQ
**Os03g0237250_OsIDD14**	.................MAAIDGHGASAVAREHLRGG................GVGAGI.
**Os01g0888700_OsIDDa**	.................KQRKRAIAAAHGRRRPISSRNAYKYKG.KGTMNSKIERQACKW
**Os01g0871200_OsIDDb**	....
**Os03g0838800_OsIDDc**	....
**Os04g0165200_ART2**	....
**Os07g0162300_OsIDDe**	.................KENKKKVKAEKREARKDKIPKAEKKKRKKMAKAKCKR......
**Os10g0419200_Ehd2**	...............RLPPVNETWSHNF................................
**Os08g0562300_OsIDDf**	....
**Os02g0572900_STOP1**	....
**Os12g0170400_DART1**	....

**Solyc01g005060_SlIDD12**	....
**Solyc01g007120**	....
**Solyc01g099340_SlIDD10**	QQQVN..............HQSIQRVNS.....IWDD................
**Solyc02g062940_SlIDD17**	AAARPFDSGEFC.................GKEFGRSS................
**Solyc02g085580_SlIDD1**	TSAGSFGSADFS.................GKDLGRNS................
**Solyc03g098070**	KLSQL..
**Solyc03g121660_SlIDD11**	.NETQSQ.......................NHKTWQS................
**Solyc04g008500_SlIDD4**	EQQQQN.......NGLNNMMSSLLDPERNQIQQSFG...SATND.........
**Solyc04g056320_SlIDDb**	....
**Solyc04g080130_SlIDD18**	AEP....WDDPS.................DRKPAML.................
**Solyc05g054030_SlIDD5**	....
**Solyc06g062670_SlIDD2**	...TEFK.......................NHKTWQS................
**Solyc06g065440**	....
**Solyc06g072360_SlIDD9**	KV...
**Solyc06g075250_SlIDD7**	ASAMGLS..........DYSETH..............................
**Solyc07g053570_SlIDD8**	....
**Solyc08g063040_SlIDD16**	AAPASFD.......QHHHHHQNQNQ.....NQLPWQN................
**Solyc08g080850**	....
**Solyc09g007550_SlIDD13**	.SAMGLS..........HYSTGH..............................
**Solyc09g065670_SlIDD15**	GAGAGAAAD....................STTGTWGDC...............
**Solyc09g074780_SlIDD3**	RLQASSS..........FHHQQNINHNSGLEKPIWDV................
**Solyc10g084180_SlIDD14**	SSAMGLS..........HLQ.................................
**Solyc11g017140**	....
**Solyc11g066420**	....
**Solyc11g069240_SlIDD6**	....
**AT1G03840_MGP/AtIDD3**	....
**AT1G14580_BLJ/AtIDD6**	G................RSGGPPLDAEMKFSHPNHPYGKA.............
**AT1G25250_FAL/AtIDD16**	....
**AT1G34370_AtSTOP1**	....
**AT1G55110_AtIDD7**	TSATEQQ.......................QQQHSHQD...............
**AT1G68130_AtIDD14**	SS...
**AT2G01940_SGR5/AtIDD15**	....
**AT2G02070_RVN/AtIDD5**	QQQQQQQQQQQHGNSRERVGSSSDSADRSSMNVNTGGGPASTSPPYGIHHASF
**AT2G02080_IME/AtIDD4**	S................ARGGVSLDGEAKFPEQNYPFGRG.............
**AT2G24990_AtIDDa**	....
**AT3G13810_AtIDD11**	SSASDQL.......................HPKPWQG................
**AT3G45260.1_BIB/AtIDD9**	TADMNQ...
**AT3G50700_AtIDD2**	MAT.TFGSGEFS.................GKDISRRKS...............
**AT4G02670_AtIDD12**	HLGFSGAHR....................M.......................
**AT5G03150_JKD/AtIDD10**	....
**AT5G22890_STOP2**	....
**AT5G37350_AtIDDb**	....
**AT5G44160_NUC/AtIDD8**	....
**AT5G60470_EGRET/AtIDD13**	KLAATIV..........GRKQP...............................
**AT5G66730_ENY/AtIDD1**	L....FGSGEFS.................GKDIGRSS................
**Os03g0197800_OsIDD1**	GSATVAAETFGAA........HGGQANPWERNPSSSPIL..............
**Os01g0195000_OsIDD2**	S.AAPYD.........HHHH..........SNKPWHS................
**Os09g0555700_OsIDD3**	AG...............L.HVGSLDPANMN.......................
**Os02g0672100_OsIDD4**	GG...............SDMHGTLEAEMKSASSSFNGGRMQ............
**Os07g0581366_OsIDD5**	SAQAPWEEA....................KRKTNGRTIL..............
**Os08g0554400_OsIDD6**	AG...............MGMAGSLDQEMK........................
**Os02g0518500_OsIDD7**	KPQEHIY..........SYHQSSLDPTA.LEKPIWDS................
**Os01g0242200_OsIDD8**	....
**Os01g0935000_OsIDD9**	....
**Os04g0566400_OsIDD10**	S................SDMS.SLEAEMKSASS.FNGRRMP............
**Os01g0572300_OsIDD11**	AGMAGYEPPPPHHGQPQHQHQQQQQQQQGGSNEPWHGMGSHS...........
**Os08g0467100_OsIDD12**	RTMAMDLN...
**Os09g0449400_OsIDD13**	HANSMGIM...
**Os03g0237250_OsIDD14**	....
**Os01g0888700_OsIDDa**	....
**Os01g0871200_OsIDDb**	....
**Os03g0838800_OsIDDc**	....
**Os04g0165200_ART2**	....
**Os07g0162300_OsIDDe**	....
**Os10g0419200_Ehd2**	....
**Os08g0562300_OsIDDf**	....
**Os02g0572900_STOP1**	....
**Os12g0170400_DART1**	....
