# Supplementary material for: Functional characterization of plant specific Indeterminate Domain (IDD) transcription factors in tomato (Solanum lycopersicum L.)
Source: Sci Rep. 2024 Apr 5;14:8015. doi: 10.1038/s41598-024-58903-0 (PMC10997639; doi:10.1038/s41598-024-58903-0)
Supplement: Supplementary file 2 — Supplementary Figure S2. [file 41598_2024_58903_MOESM2_ESM.pdf]

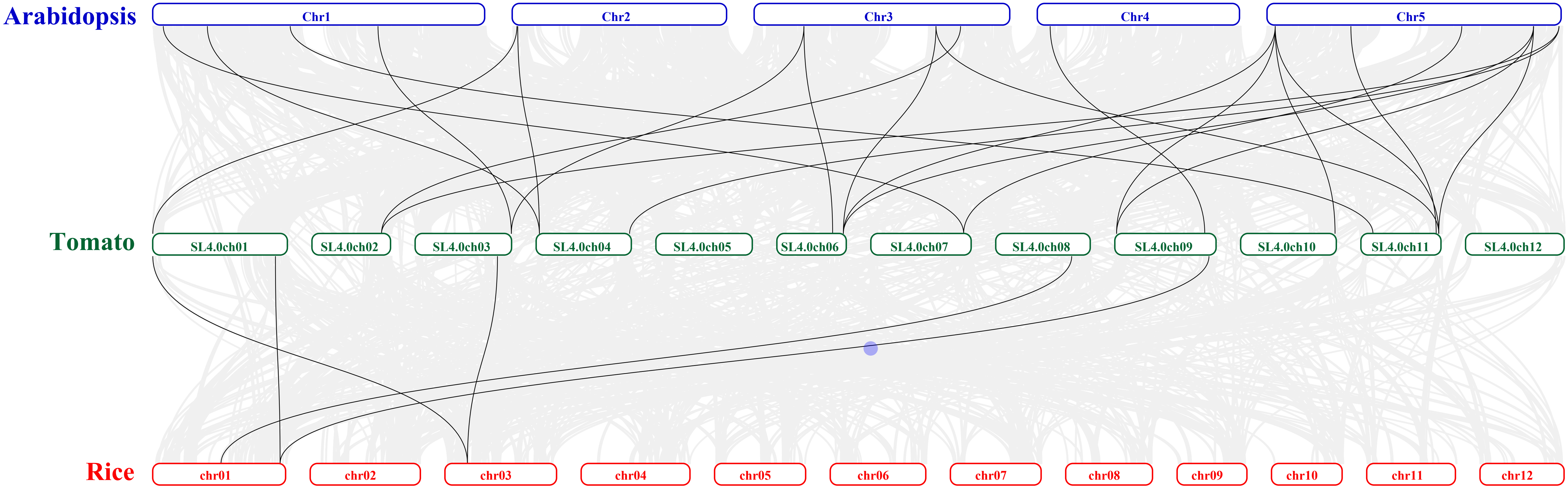

Figure S2. The synteny analysis of SlIDD family genes. Synteny analysis of IDD genes between rice, tomato, and Arabidopsis. Gray lines: all collinear blocks within tomato and other plant genomes. Red lines: the synteny of IDD gene pairs. The species names with the prefixes Os, Sl, and At indicate rice, tomato, and Arabidopsis, respectively.
