# Supplementary material for: Functional characterization of plant specific Indeterminate Domain (IDD) transcription factors in tomato (Solanum lycopersicum L.)
Source: Sci Rep. 2024 Apr 5;14:8015. doi: 10.1038/s41598-024-58903-0 (PMC10997639; doi:10.1038/s41598-024-58903-0)
Supplement: Supplementary file 3 — Supplementary Figure S3. [file 41598_2024_58903_MOESM3_ESM.pdf]

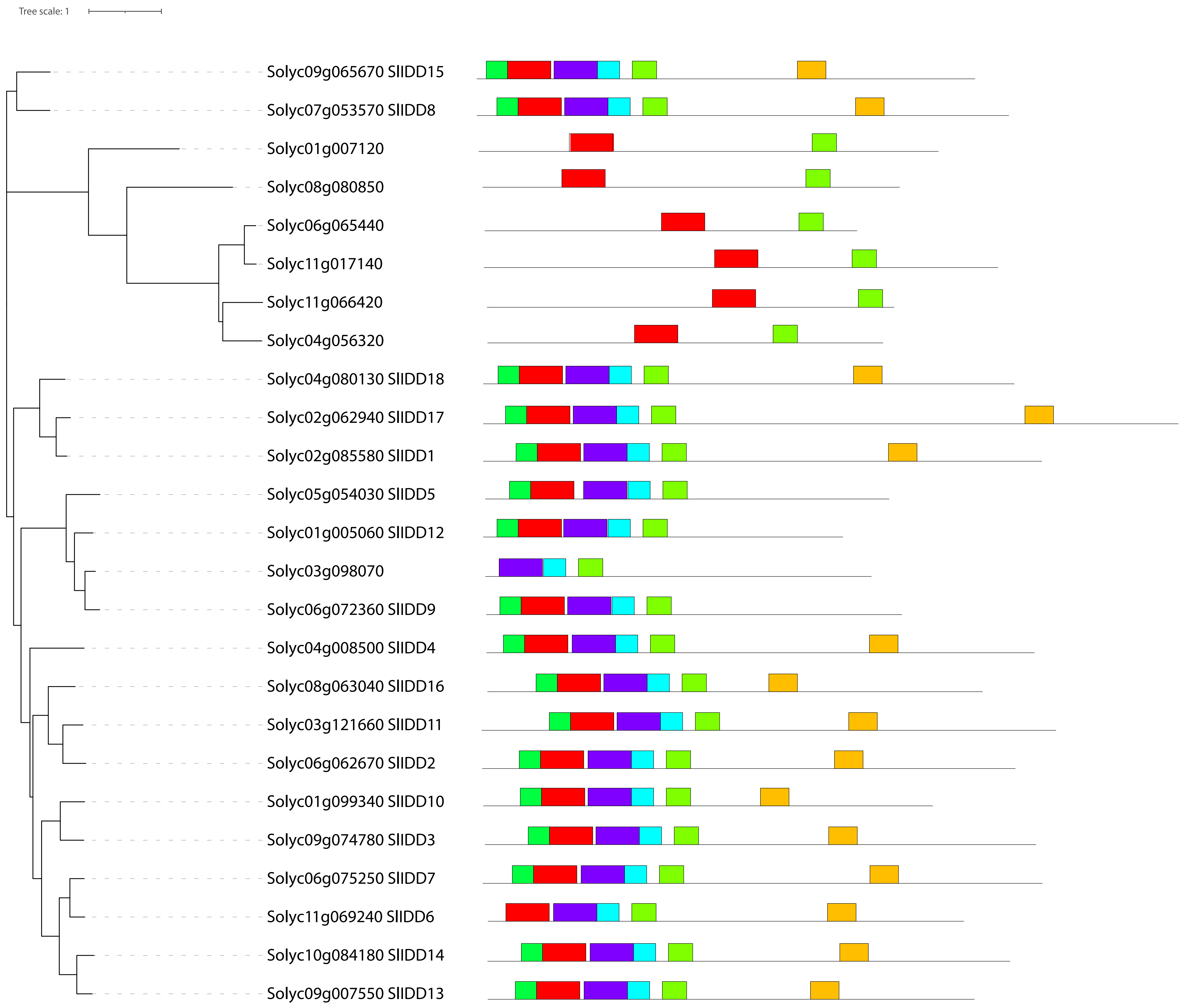

Figure S3. Motif analysis of Tomato IDD-like transcription factors. Sequences with 4 conserved C-terminus motifs were named as *IDD*s.
